# Organosilicons of different molecular size and chemical structure as consolidants for waterlogged archaeological wood – a new reversible and retreatable method

**DOI:** 10.1038/s41598-020-59240-8

**Published:** 2020-02-10

**Authors:** Magdalena Broda, Izabela Dąbek, Agnieszka Dutkiewicz, Michał Dutkiewicz, Carmen-Mihaela Popescu, Bartłomiej Mazela, Hieronim Maciejewski

**Affiliations:** 1 Poznań University of Life Sciences, Faculty of Wood Technology, Department of Wood Science, Wojska Polskiego 28, 60-637, Poznań, Poland; 2 BioComposites Centre, Bangor University, Gwynedd, LL57 2UW UK; 3 Adam Mickiewicz University Foundation, Poznań Science and Technology Park, Rubież 46, 61-612, Poznań, Poland; 4 Centre for Advanced Technologies, Adam Mickiewicz University, Uniwersytetu Poznańskiego 10, 61-614, Poznań, Poland; 5 Petru Poni Institute of Macromolecular Chemistry of the Romanian Academy, 41A Grigore Ghica Voda Alley, 700487 Iasi, Romania; 6 Poznań University of Life Sciences, Institute of Wood Chemical Technology, Faculty of Wood Technology, Wojska Polskiego 28, 60-637, Poznań, Poland; 7 Faculty of Chemistry, Adam Mickiewicz University, Uniwersytetu Poznańskiego 8, 61-614, Poznań, Poland

**Keywords:** Materials chemistry, Materials science

## Abstract

Ineffectiveness of the chemicals applied so far for waterlogged wood conservation created the need to develop new more, efficient and reliable agents. As an alternative, a new method with the use of organosilicon compounds differing in chemical composition and molecular weight has been investigated. The results obtained show the potential of organosilicons as consolidants in waterlogged wood conservation able to effectively stabilise wood dimensions upon drying. The best wood stabilisers were low-molecular organosilicons enable to penetrate the cell wall as well as chemicals with functional groups capable of interacting with wood polymers and forming stabilising coatings on the cell wall surface. The best anti-shrink efficiency values were obtained for (3-Mercaptopropyl)trimethoxysilane, (3-Aminopropyl)triethoxysilane, 1,3-Bis(3-aminopropyl)-1,1,3,3-tetramethyldisiloxane, reaching 98, 91 and 91%, respectively. Most of the applied organosilicons reduced wood hygroscopicity, which limits the risk of further dimensional changes of wood exposed to a variable air moisture content and potentially reduces wood biodegradation. In the light of our studies, the proposed method of waterlogged wood conservation with organosilicons is potentially reversible in the case of siloxanes and amino-silanes as well as retreatable, which complies with the requirements of the conservation ethics.

## Introduction

Wooden Cultural Heritage constitutes priceless evidence of human history. Therefore, to pass on our culture and traditions to future generations and help forge the sense of identity of modern societies, we are obliged to ensure its proper protection and conservation. Since the recent research revealed that the most popular consolidants used for archaeological waterlogged wood, including alum salts and polyethylene glycol, occurred ineffective over time and could even pose a threat for the integrity of valuable wooden relics, a requirement for developing new more efficient and reliable agents has emerged, addressing particular interest of scientists and conservators^1–7^.

Recent research on alternative consolidants for waterlogged archaeological wood focuses on natural, bio-friendly agents compatible with wood structure, e.g. chitosan^8,9^, guar gum^9^, cross-linkable cellulose ethers^10^, nanocomposites based on esterified colophony and halloysite clay nanotubes^11^ or beeswax and halloysite nanotubes^12^, functionalised oligoamides^13^, lignin-like oligomers^14,15^ or lignin–silicone hybrid materials^16^. There is also another approach employing the activator regenerated by electron transfer atom transfer radical polymerisation (ARGET ATRP) method for graft polymerisation of some chemicals after immobilisation of the initiator (2-bromoisobutyryl bromide) on a wood substrate^17^. Furthermore, some new multi-functional systems are being developed providing consolidation and simultaneous deacidification of wood, such as nanocomposites made of propylene glycol-modified silane and nanostructured calcium hydroxide^18^ or polyethylene glycol and halloysite nanotubes containing calcium hydroxide^19^, as well as a supramolecular polymer network system based on functionalised natural polymers (guar gum, chitosan) that faces simultaneously three different issues connected with waterlogged wood: instability on drying, chemical degradation promoted by Fe^3+^-catalysed synthesis of acids and biological degradation^20^.

The critical issue of waterlogged wood is its dimensional instability upon drying. Since the wooden cell walls become weakened due to degradation, they get prone to collapse under capillary forces of evaporating water when the excavated object is exposed to air, which results in its shrinkage and cracking^21,22^. Therefore, consolidation agents dedicated to waterlogged wood should primarily provide its integrity and efficient dimensional stabilisation to protect original appearance of historical artefacts and prevent their permanent destruction. They should also protect the wooden object against further dimensional changes (swelling or shrinkage), as well as biotic and abiotic degradation, and maintain the mechanical strength of wood. Since these issues are closely related to wood hygroscopicity^23–27^, its reduction by conservation agents seems essential. Furthermore, consolidants should be chemically stable, ageing-resistant, compatible with wood structure, and preferably bio-friendly and cost-efficient. Conservation ethics also impose desirability for reversibility of the conservation treatment and the possibility for further re-conservation by using different agents.

Organosilicon compounds shall potentially comply with most of the above-mentioned requirements. They are characterised by high thermal stability and chemical resistance to external factors (humidity, UV radiation), thereby have been applied in many industry sectors, including polymer, textile, building materials, pulp and paper, cosmetic, medical, pharmaceutical and food production^28–31^. The most common silicon derivatives are organofunctional alkoxysilanes mainly applied as binding agents, adhesion promoters and surface modifiers^32^. Due to their unique bifunctional structure and the resulting specific reactivity, they have also been utilised in preservation and conservation of wood and wood-based products, serving as additives for preservatives, improving weathering performance, decreasing wood hydrophilicity, reducing flammability and enhancing decay resistance^33–35^. They contain readily hydrolysable alkoxy groups as well as an organic functional group which makes them possible to impart specific functions to the modified material^30,33^. The polymerisation of alkoxysilanes, called the sol-gel process, proceeds through a series of consecutive hydrolysis and condensation reactions^36,37^. The amount of water contained in waterlogged wood should be sufficient to activate silane molecules and allow them to condense and chemically bind to the wood matrix without using a catalyst. The hydrolysed alkoxy groups can react with other silane molecules establishing Si-O-Si bonds in the condensation process. Simultaneously, they can also interact with hydroxyl groups present on wood polymers forming siloxy bonds (Si-O-C) between organosilicon and wood^29,35–37^. A resulting spatial network made of polysiloxane and the wood polymers could potentially enhance and stabilise the weakened cell walls of degraded waterlogged wood. However, it is worth noting that the siloxy bonds formed due to the condensation reaction can undergo secondary hydrolysis. Therefore, to obtain a more durable wood modification, silicon derivatives (e.g. isocyanate or epoxy) containing chemical groups capable of forming more stable covalent bonds with hydroxyls present on wood polymers, can also be applied^38^.

Apart from organofunctional silanes, modification of wood can be performed using functionalised polysiloxanes. They are characterised by a stable and flexible siloxane chain that enables a proper orientation on the modified surface. Moreover, various functional groups can be attached to the siloxane chain, allowing the formation of stable linkage with a substrate and giving it specific properties^39^.

Although recognised as potential consolidants for waterlogged wood in the 90s^40,41^, silicones have not so far been commonly used in conservation mainly for economic reasons. The current research has shown the effectiveness of methyltrimethoxysilane and a mixture of silicone oil with methyltrimethoxysilane or isobutyltrimethoxysilane in the stabilisation of waterlogged wood dimensions^40,42,43^. Our preliminary study also revealed a good stabilising and hydrophobising effect of some alkoxysilanes, including Methyltrimethoxysilane and (3-Mercaptopropyl)trimethoxysilane, and 1,3-Bis(diethylamino)-3-propoxypropanol)-1,1,3,3-tetramethyldisiloxane on degraded waterlogged wood^44–46^. The ability of silicones to penetrate wood tissue and possibility to bulk the cell wall, their longevity, ease of treatment and curation, reasonably short timescale and potential reversibility of the conservation process make them an interesting consideration for the treatment of waterlogged wood and may serve as an alternative set of utilities in the conservator’s tool kit. For the above reasons, we decided to explore in depth the potential of organosilicons for the conservation of waterlogged wooden artefacts.

For this research, a set of silanes and siloxanes differing in molecular size and chemical structure were synthesised to investigate their efficiency in the stabilisation of waterlogged wood dimensions during drying. The study aimed at assessing the most effective chemical structure that ensures the best dimensional stabilisation of highly degraded waterlogged wood and determining the influence of organosilicons on wood moisture properties. To comply with conservation ethics, the potential reversibility and re-treatability of the method were examined.

## Results and Discussion

### Effectiveness of the treatment

**Figure 1:**
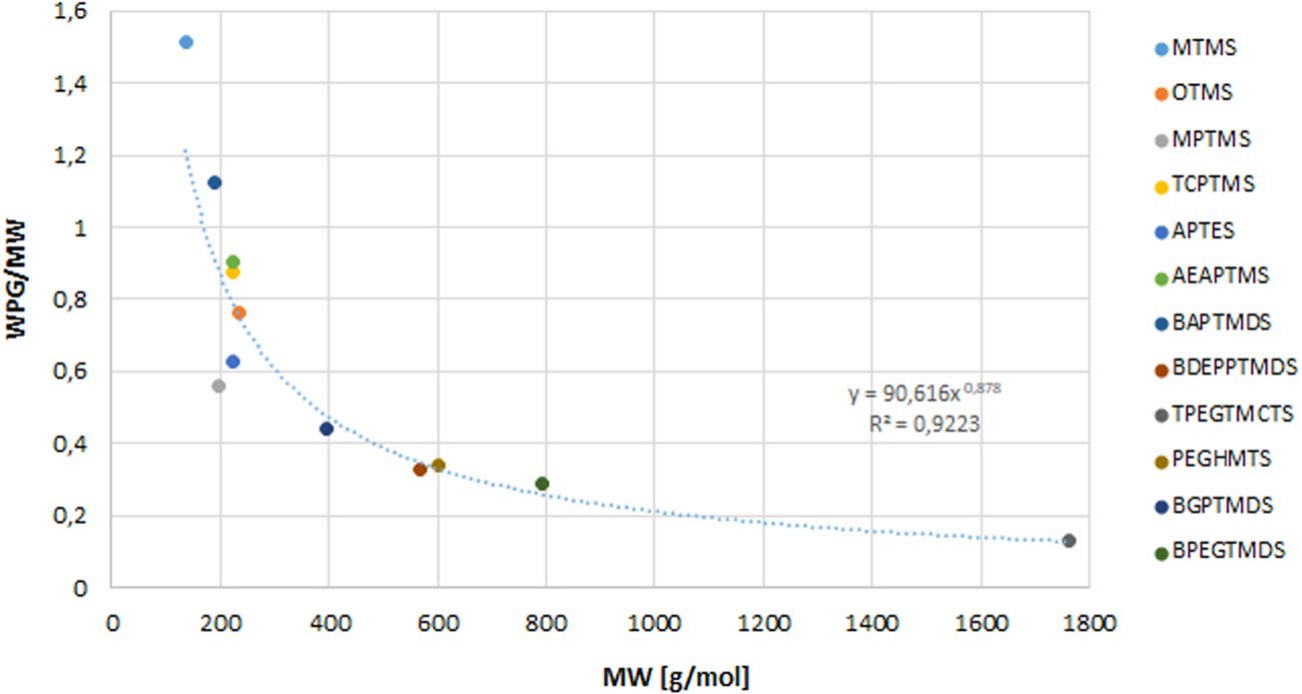
The correlation between the molecular weight of organosilicon particles and their relative number (expressed as a ratio of WPG to MW) absorbed in wood.

To evaluate the treatment effectiveness, weight percent gain for treated waterlogged wood samples was calculated (see Table 1). The increased masses of the samples confirm an efficient impregnation of wood by the applied chemicals, whereby the correlation between the molecular weight of organosilicon particles and their relative number absorbed in wood can be seen (Fig. 1). Generally, the smallest was the molecular weight of the applied derivatives, the easier they penetrated wood, which is related to the porosity of the wooden cell wall^47^.

**Table 1:** The physical parameters measured or calculated for particular organosilicons or wood samples treated with them: S_v_ – volumetric shrinkage of wood, ASE_v_ – anti-shrink efficiency for particular chemicals, WPG – weigh percent gain, MW – molecular weight of organosilicon monomer, MC – moisture content in wood sample.

Sample ID (organosilicone applied)	S_v_ [%]	ASE_v_ [%]	WPG [%]	MW [g/mol]	MC [%]
MTMS	9.9 ± 1.5	81.1 ± 2.8	206.1 ± 10.5	136.22	5.0
OTMS	15.2 ± 2.4	71.0 ± 4.5	179.0 ± 23.8	234.41	2.6
MPTMS	1.1 ± 1.9	97.9 ± 3.6	109.9 ± 15.4	196.34	2.6
TCPTMS	16.5 ± 2.6	68.5 ± 5.0	193.4 ± 11.9	221.35	2.2
APTES	4.6 ± 0.9	91.1 ± 1.7	138.3 ± 5.4	221.37	7.8
AEAPTMS	15.1 ± 0.3	71.1 ± 0.7	201.6 ± 6.4	222.36	8.9
BAPTMDS	4.8 ± 1.6	90.8 ± 3.1	212.1 ± 14.2	188.38	7.9
BDEPPTMDS	5.3 ± 3.2	89.8 ± 6.0	185.6 ± 8.8	568.61	2.1
TPEGTMCTS	27.1 ± 3.9	48.2 ± 7.5	229.1 ± 21.3	1762.40	2.5
PEGHMTS	6.0 ± 2.6	88.5 ± 5.0	203.2 ± 6.1	602.76	2.6
BGPTMDS	17.5 ± 2.0	66.5 ± 3.9	174.5 ± 31.8	394.74	1.8
BPEGTMDS	17.5 ± 2.7	66.5 ± 5.1	226.9 ± 13.9	793.05	2.2
CE	52.3 ± 5.2	—	—	—	6.6

**Figure 2:**
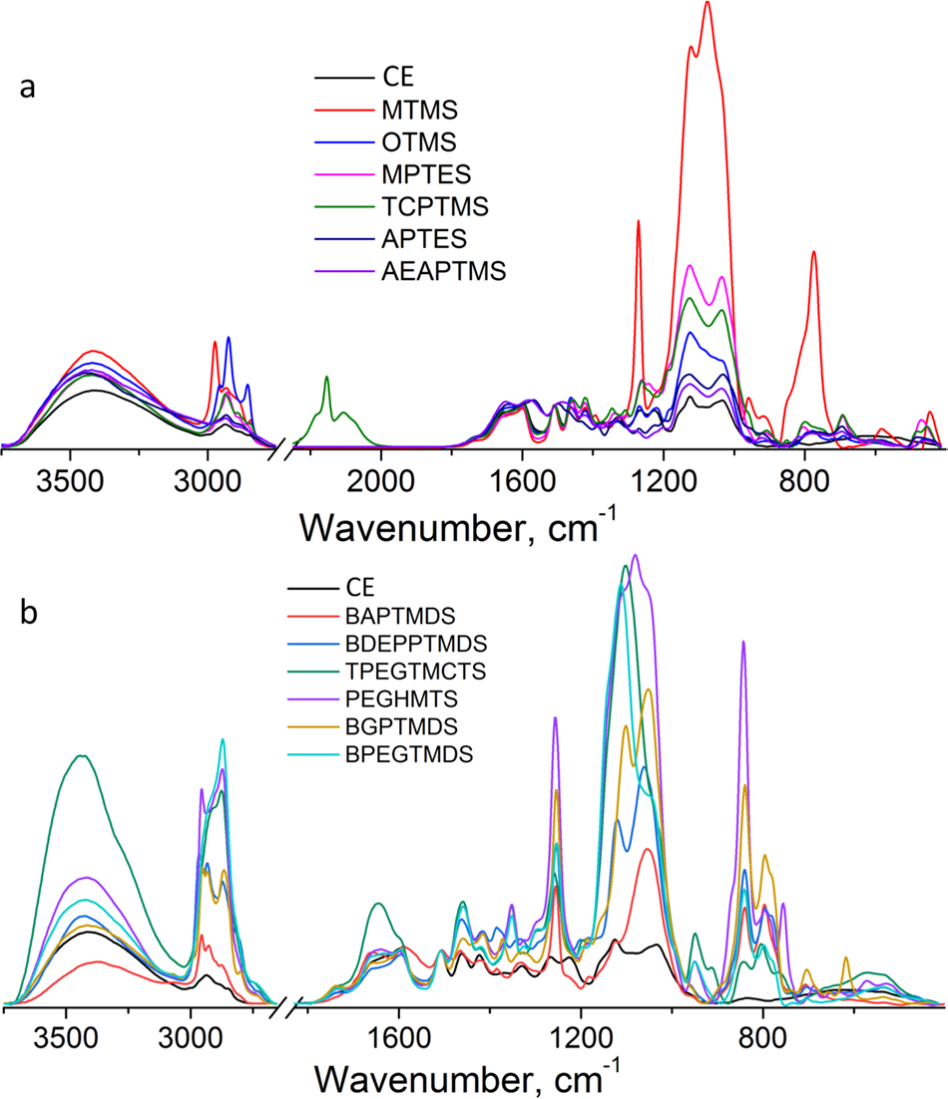
FT-IR spectra of silane- (**a**) and siloxane-treated (**b**) waterlogged elm samples.

To confirm the presence of organosilicons inside the treated wood samples, FT-IR analyses were performed. Infrared spectra of untreated (CE) and silane/siloxane-treated samples are presented in Fig. 2.

Comparing to control spectrum, the spectra of treated wood indicate, apart from the bands assigned to wood, the presence of the bands which are expected to appear due to the chemical bonds from the silanes/siloxanes, indicating the presence of the chemicals onto the wood.

MTMS, OTMS, MPTES and TCPTMS spectra present higher intensities for the bands between 3000–2800 cm^−1^. In the fingerprint region, new bands at around 1270, 1169, 1128, 1076, 958, 813, 772, 690 and 447 cm^−1^ were identified. Both the band intensities and positions vary for each spectrum. In order to highlight the differences appearing between the spectra of control and treated samples, the second derivative spectra were plotted (Figs. S1a and S1b in Supplementary Information) and the main bands were marked with arrows.

Concerning the MPTES, the most informative bands are observed at 1340 and 1308 cm^−1^ due to the existence of a larger number of –CH_2_ groups in the solid network, similar to OTMS and TCPTMS. Moreover, TCPTMS, present bands at 2187, 2154, 2107 and 2072 cm^−1^.

APTES and AEAPTMS spectra present new identifiable bands (apart of those listed above for the other silane-treated samples) at 1645/1653, 1563/1568, 1385, 1347 and 1331/1323, and 1155/1152 cm^−1^.

The intensities of the spectral bands assigned to the silane component are well correlated with the WPG, higher values of WPG being reflected in higher intensities of the wood treated spectra.

The spectra of the samples treated with siloxanes are more complex and clearly indicate their presence in the wood. BAPTMDS spectrum shows bands at 2957, 2925, 2896, 2870 and 2795 cm^−1^. They are shifted to lower values comparing to control sample, indicating the strong presence of the CH_2_/CH_3_ groups from BAPTMDS. In the fingerprint region, the presence of BAPTMDS is indicated by increased intensity and shift to the higher wavenumber of the bands from 1666, 1336 cm^−1^, sharp and high-intensity band at 1256 cm^−1^, new doublet from 840/796 cm^−1^, and 1042 cm^−1^ overlapped with the bands for wood from 1053 and 1029 cm^−1^. New bands were identified at 1568, 1384, 1301, 1183, 1003 cm^−1^. For the BDEPPTMDS, new bands were identified at 1201, 1062, and 1334 cm^−1^. TPEGTMCTS spectrum presented particular bands at: 1353, 1196, 1142, 1103, and 1025 cm^−1^, while PEGHMTS spectrum present very high intensities between 3000–2700 cm^−1^, and bands at 1352, 1257 cm^−1^, triplet from 1119, 1081, 1041 cm^−1^, and at 842, 790 and 755 cm^−1^. BGPTMDS and BPEGTMDS spectra present similar bands with the other siloxane treated samples.

A complete list of band positions and their assignment belonging to main groups in the wood, as well as in the silane/siloxane-treated wood samples are presented in the Tables S1 and S2 (see Supplementary Information).

Organosilicon penetration into wood samples was also confirmed by SEM-EDX images with Si mapping on the cross-sections through the middle of the samples (see Figs. S27–S30 in Supplementary Information).

### Dimensional stability

**Figure 3:**
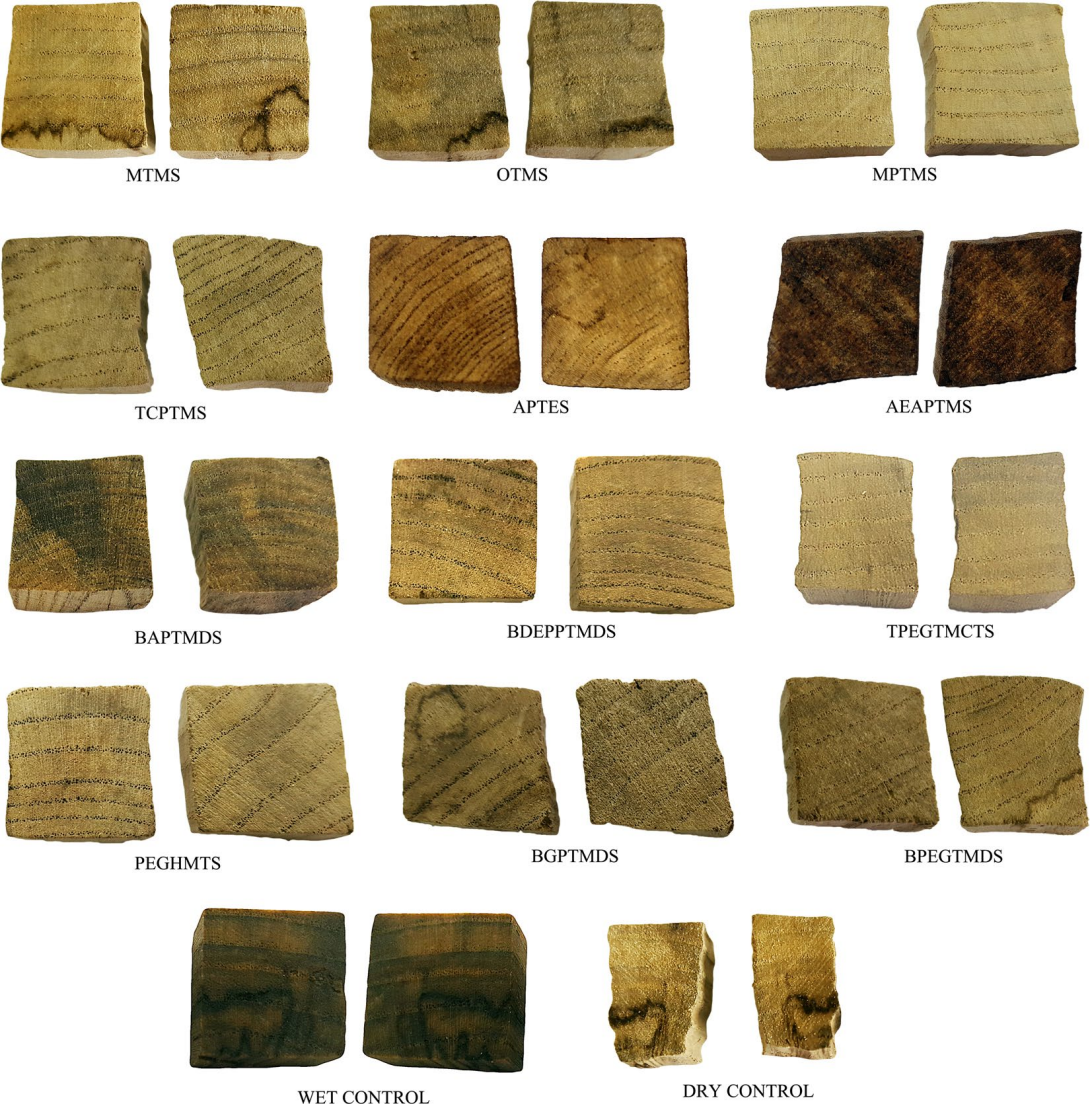
The overall appearance of the air-dried treated waterlogged elm samples and untreated wood – wet and air-dried (the dark stains on some specimens (MTMS, OTMS, BGPTMDS, BPGTMDS, control) occurred naturally in wood result from decay at the deposition site, not from the treatment applied).

The applied treatment with organosilicons resulted in stabilisation of waterlogged wood dimensions. The post-treatment colouration and overall appearance of exemplary treated/untreated and air-dried samples are presented in Fig. 3. The results expressed as a volumetric shrinkage (S_v_) of wood samples and anti-shrink efficiency (ASE) are presented in Table 1.

Generally, wood treatment with chemicals with the smallest molecules (MTMS, BAPTMDS, MPTMS) resulted in a good dimensional stabilisation with S_v_ between 1 and 10% (for comparison – untreated samples shrunk by 52%), which may be explained by their ability to penetrate the cell wall thus enhancing its structure. For bigger molecules, the less stabilisation effect was observed, with S_v_ reaching 27% for wood treated with high-molecular TPEGTMCTS. However, the correlation between molecular weight (MW) of organosilicons and their stabilisation efficiency is not direct, and certain derogations from this rule are noticeable, which results from their specific chemical composition – the presence of particular reactive groups or the length of side chains (Fig. 1).

Inherently, the monomers of all the silanes applied not only can condense with one another, but they can also bind to wood polymers via alkoxy bonds acting as cross-linkers. As a result, a new composite made of silane and wood polymers is formed within the cell wall, stabilising the wood structure. However, the alkoxy bonds are unstable, and the whole polymer network can rearrange due to their alternate hydrolysis and condensation^29,48^. In the case of the smallest MTMS molecules able to penetrate the cell wall with high efficiency, this reactivity and the filling of the cavities/pores in the cell wall seem sufficient to enhance wood structure and effectively stabilise its dimensions (S_v_ of 10%), while for silanes with longer side chains (OTMS, AEAPTMS), which may have difficulties with penetration of the cell wall and formation of a regular spatial network due to their dimensions, it is not enough to provide proper stabilisation of wood (S_v_ of about 15%). Only the presence of additional reactive groups, providing supplementary chemical interactions with wood polymers, results in better stabilising effect. The MPTMS, for which the presence of –SH group and its ability to form stable covalent chemical bonds with wood polymers (C–S–C), enables the creation of a sustained stabilising spatial network inside the cell wall, reinforcing its structure and preventing collapse, which results in wood S_v_ of only 1.1%^45,49^. Although TCPTMS has a –SCN group, enabling similar interactions with wood polymers, its stabilising effectiveness is much lower (S_v_ of 16.5%). Perhaps –SCN group is a steric hindrance that inhibits the formation of a regular stabilising polymer network, similar to long side chains of OTMS and AEAPTMS. In the case of APTES (S_v_ of 4.6%), except the interactions via alkoxy bonds, the good stabilising effect is likely due to additional hydrogen bonds that may be formed between amino groups and –OH groups of wood components.

When considering the stabilising effect of the siloxanes used, the role of their molecular size and the presence of reactive groups is apparent, as for silanes described above, resulting in S_v_ of 4.8% for wood impregnated with low-molecular BAPTMDS with amino groups and S_v_ of 5.3% for samples treated with BDEPPTMDS containing nitrogen atoms. The only startling example is BGPTMDS containing epoxy groups which were supposed to act as cross-linkers between wood polymers thus enhance the wood structure, but apparently, the dimensions of the siloxane molecules prevented the formation of a stabilising wood-polymer network, resulting in wood shrinkage of 17.5%. On the other hand, the high effectiveness of high-molecular PEGHMTS (S_v_ of 6%) was also surprising. Possibly the spatial structure of its molecules can play a role in enhancing a wood structure.

Shrinkage of treated wood translates into anti-shrink efficiency (ASE), which indicates a percentage of the shrinkage of untreated wood that has been suppressed by the applied treatment. In a wood conservation practice, ASE values exceeding 70% are often considered acceptable^50^. From this point of view, six out of ten tested organosilicons could be potentially useful for waterlogged wood conservation, whilst for three of them (MPTMS, APTES and BAPTMDS) ASE was over 90%.

**Figure 4:**
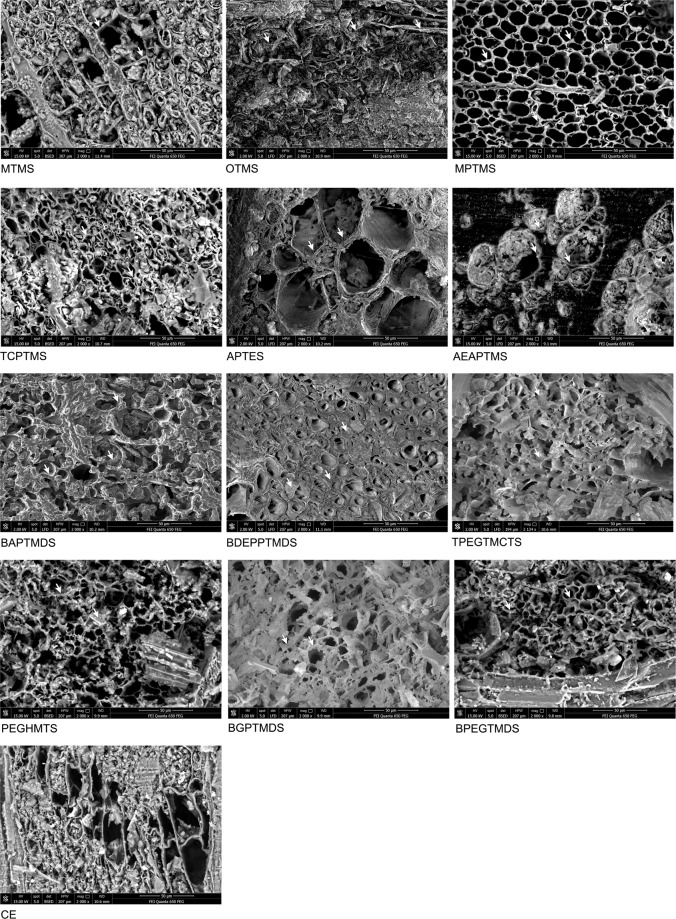
SEM images of air-dried untreated and treated waterlogged elm samples.

SEM analysis of the treated and untreated samples (Fig. 4) confirms the results of dimensional stability described above. The image of highly shrunken CE reveals an irregular shape of the flattened cells with the thin cell walls full of uneven cracks and distortions. The most effective treatment with MPTMS, APTES, BAPTMDS resulted in a more regular shape of the cells with the thicker cell walls alike in fresh non-degraded wood. Comparison of the SEM images shows a different way of deposition of particular organosilicons in wood: low-molecular alkoxysilanes, like MTMS, MPTMS or TCPTMS, seem to penetrate the cell wall or form a very thin layer on its surface, while high-molecular silanes and siloxanes cover the cell walls with a thick coating or even fill the cell lumina, which apparently not in all cases is sufficient for proper preservation of wood structure. This, together with different stabilising effectiveness of particular organosilicons, indicates a different mechanism of wood stabilisation dependent on their chemical composition.

### Moisture properties

A different chemical structure of the organosilicons used and the consequent diverse hydrophobicity translated into moisture properties of the treated wood samples. Generally, the applied treatment resulted in a decrease in wood moisture content (Table 1) to 5% for MTMS and 2–2.5% for other chemicals. The only exception was for amino-silicons that increased wood MC to about 8–9%, which means that they increased natural hygroscopicity of the degraded elm (MC of untreated wood was 6.6%). The hydrophobising effect of most of the applied organosilicons limits the possibility for further changes in wood dimensions due to the changes in an air moisture content, important from the conservation perspective. It should also contribute to the reduction of fungal activity in the treated wood, limiting its biodegradation. On the other hand, the hydrophilic effect of amino-silicons may explain their efficiency in the stabilisation of wood dimensions since they can retain more water molecules inside wood, keeping the cell walls in a more swollen state thus preventing to some extent their collapse or shrinkage. It is also known that chemicals of this type limit degradation of wood by decaying fungi^33,51,52^.

**Figure 5:**
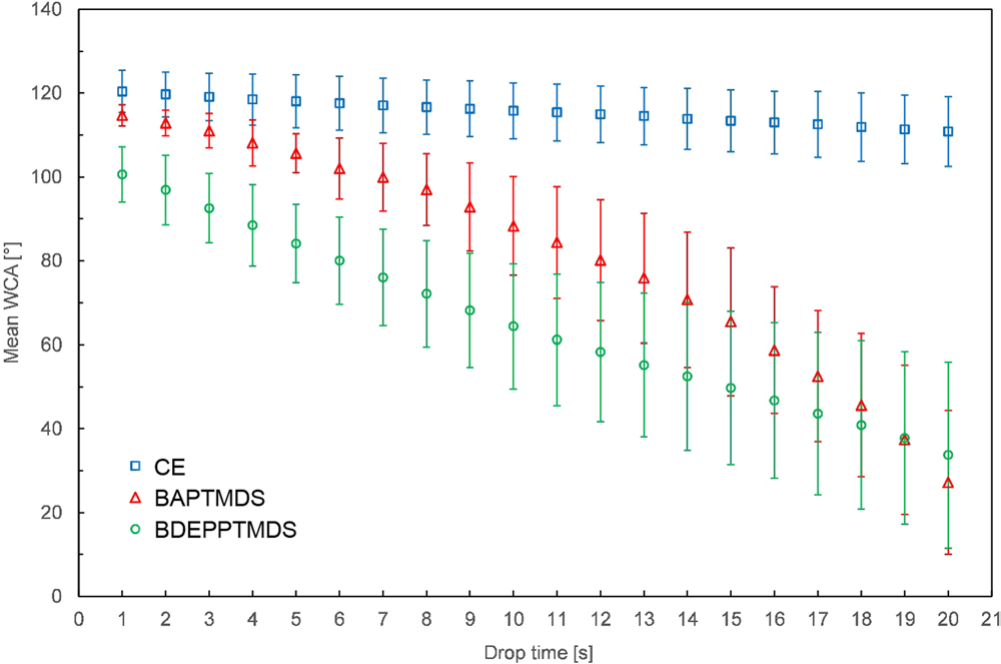
Time-resolved WCA values for wood samples treated with amino-functional disiloxanes.

Along with hygroscopicity, also wettability of the treated wood was examined by means of the water contact angle measurement (Table 2 and Fig. 5).

**Table 2:** Results of the water contact angle (WCA) measurements for untreated and treated wood.

Entry	Sample ID	WCA [°]
1	MTMS	142.2 ± 0.6^r^
2	OTMS	137 ± 2.98
3	MPTMS	133.7 ± 0.69
4	TCPTMS	139.1 ± 2.4
5	APTES	138.4 ± 2.77
6	AEAPTMS	132.8 ± 2.14
7	BAPTMDS	79.7 ± 12.87^qs (112)^
8	BDEPPTMDS	62.6 ± 16.66^qs (100)^
9	TPEGTMCTS	−^nm^
10	PEGHMTS	−^nm^
11	BGPTMDS	128.4 ± 3.11
12	BPEGTMDS	−^nm^
13	CE	115.5 ± 6.78^ss^

^ss^– The drop slowly soaks into the wood sample after deposition.

^qs()^– The drop quickly soaks into the wood sample after deposition. First measured WCA value is given in bracket.

^nm^– Not measured, the drop quickly soaks into the wood sample during deposition.

^r^– The drop runs off the surface of the wood sample after deposition.

In general, the applied treatment with silanes resulted in a contact angle increase from 115° to 133–142° for silane-modified wood (Table 2, entries 1–6). This can be explained by the durable hydrophobic layer formation on the wood surface based on the hydrolysis of alkoxy groups present in applied silanes structure to the silanol ones and their further self-condensation with a formation of siloxane network and simultaneous bonding to the hydroxyl groups present on the wood surface (silylation). In the case of wood samples treated with siloxanes (except the BGPTMDS), the measured values of WCA decreased in comparison with untreated wood or the measurement was impossible due to immediate soaking of water drops into the wood. This phenomenon results from the lack of reactive alkoxy groups capable for the formation of durable siloxane network and simultaneous bonding to the wood surface, as well as from the presence of strongly hydrophilic polyether chains (Table 2, entries 9, 10 and 12) or amino groups (Table 2, entries 7 and 8). The relatively high WCA value obtained for the BGPTMDS-modified sample (Table 2, entry 11) can be explained by the presence of two terminal epoxy groups which, as it is commonly known, can react under acidic or basic conditions with hydroxyl groups of the cellulose-based materials in the epoxy ring-opening process, leading to siloxane bonding to the wood surface and formation of a relatively durable water repellent layer.

**Figure 6:**
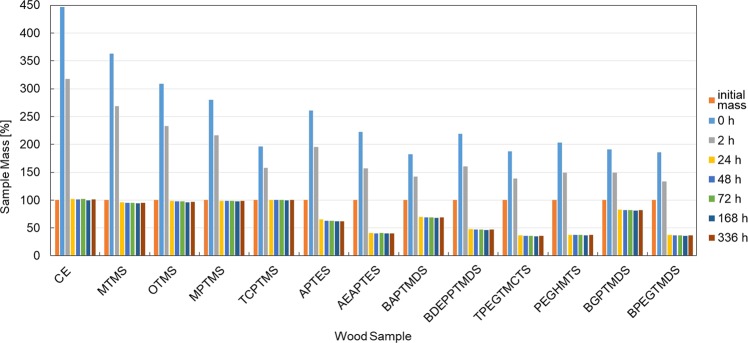
Comparison of impregnated wood samples mass before and after leaching test.

Although the mean WCA values measured for BAPTMDS and BDEPPTMDS-modified samples were much below 90° (Table 2, entries 7 and 8), which qualifies them as hydrophilic, it should be mentioned that the starting WCA values measured for deposited drops were much higher (about 100 and 112° respectively). As shown in Fig. 6, they decreased in time to get values below 40° after 20 seconds from drop deposition as a result of water drops soaking. This can be explained by the possible partial cellulose amination with BAPTMDS or weak hydrogen bonding interactions between BDEPPTMDS and –OH groups of cellulose, leading to the formation of impermanent and water permeable layers on the surface of the discussed samples.

All the above-mentioned explanations and assumptions seem to be consistent with and confirmed by the results described below concerning the reversibility of wood treatment.

### Reversibility of the treatment

Comparison of the difference between the initial mass of the sample and its mass after soaking and drying enabled to indicate the compounds which are susceptible to leaching thereby giving the possibility to reverse the treatment, and those which allow permanent wood protection (Fig. 6).

It is clear from Fig. 6 that the only relatively permanent impregnation was this with the use of hydrophobic alkoxysilanes: MTMS, OTMS, MPTMS and TCPTMS. This phenomenon can be explained by the ability to create bonds with wood polymers via alkoxy groups (Si–O–C) and stable S–C and C–N–C bonds in the case of MTTMS and TCPMS, respectively, the formation of durable siloxane (Si–O–Si) layer on the wood surface due to condensation as well as by the presence of hydrophobic functional groups, which prevent the solution from penetrating the samples and washing the protective coatings. In turn, all the siloxanes applied, as well as alkoxysilanes with amino groups, have proved to be susceptible to leaching (with the mass loss of the samples after leaching from 32 to 65%). It results from strongly hydrophilic nature of these chemicals and, in the case of siloxane derivatives, also from the lack of hydrolysable alkoxysilyl groups in their structure, which disabled the formation of covalent bonds with the wood surface. Such chemical composition enabled the water/ethanol solution to penetrate the treated samples and to elute the agents used, making the treatment reversible.

It can also be observed that the mass of the samples stabilised after 24 h of drying and only minor changes were observed for the next 13 days.

To confirm the results obtained on the basis of the mass analysis, dry wood samples before and after leaching were analysed using the FT-IR ATR technique (Table S3 in Supplementary Information). A comparison of infrared spectra recorded for the samples treated with MTMS, OTMS, MPTMS and TCPTMS before and after soaking unambiguously confirm the retention of all the bands characteristic for the structural elements of silanes used for their impregnation (see Figs. S15, S16, S18 and S22 in Supplementary Information). For the rest of the measured samples, a prominent decrease in characteristic bands intensity was observed, what is also consistent with the previous observations based on the results of the mass analysis (see Supplementary Information).

### Possibility of re-conservation

Considering the results of SEM analysis and the leaching test, it can be stated that the treatment with all the organosilicons tested provides the possibility of further re-conservation. The most durable treatment with alkoxysilanes MTMS, OTMS, MPTMS leaves the cell lumina empty thus open for further impregnation with any other chemicals, while the rest of the organosilicons applied are both easy to remove (at least partially) by simple elution with an ethanol solution and do not fill the cell lumina completely, leaving some space for other conservation agents. Therefore, the proposed method of waterlogged wood conservation with the selected organosilicon compounds complies with the requirements of the conservation principles.

## Conclusions

The results of the presented research clearly show the potential of organosilicons in waterlogged wood conservation. The most effective stabilisers of waterlogged wood dimensions were MPTMS, APTES, BAPTMDS, BDEPPTMDS, PEGHMTS and MTMS. Different molecular weight and chemical structure of these chemicals suggest a different stabilisation mechanism. The results obtained points to the conclusion that the best wood stabilisers are low-molecular organosilicons enable to penetrate the cell wall thus enhancing its structure by filling the voids, as well as chemicals with functional groups capable of interacting with wood polymers via covalent or hydrogen bonds and forming stabilising coatings on the cell wall surface.

Except for amino-silicons, all the applied silanes and siloxanes reduced natural wood hygroscopicity, which is desirable from a conservation point of view since it limits further dimensional changes of wood exposed to a variable air moisture content and potentially reduces wood biodegradation.

The proposed method of waterlogged wood conservation is potentially reversible in the case of application of siloxanes and amino-silanes as suggested by the study. But more importantly, it also turned out to be retreatable, since even the most durable treatment with alkoxysilanes leaves the cell lumina empty and feasible to further re-filling with different conservation agents.

Summarising, the investigated method of waterlogged wood conservation with the selected organosilicon compounds, although it requires further research on its long-term effectiveness and reliability, seems promising and adheres to the principles of the conservation ethics.

## Methods

### Materials

New conservation treatment was tested using waterlogged elm (*Ulmus* sp. L.) excavated from the bottom of the Lednica Lake in the Wielkopolska Region, Poland. About a thousand-year-old wood was highly degraded, with loss of wood substance estimated at c.a. 70%^44^.

### Organosilicon synthesis

**Figure 7:**
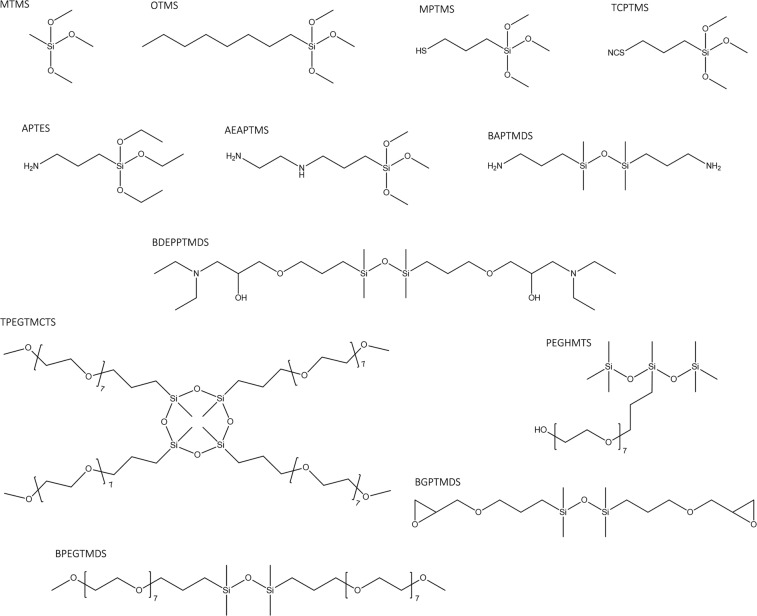
The structural formulas of the applied silanes and siloxanes.

The following organosilicons were used for the treatment (Fig. 7):

- Methyltrimethoxysilane (MTMS
- N-Octyltrimethoxysilane (OTMS)
- (3-Mercaptopropyl)trimethoxysilane (MPTMS)
- (3-Thiocyanatopropyl)trimethoxysilane (TCPTMS)
- (3-Aminopropyl)triethoxysilane (APTES)
- N-[(2-Aminoethyl)3-aminopropyl]-trimethoxysilane (AEAPTMS)
- 1,3-Bis(3-aminopropyl)-1,1,3,3-tetramethyldisiloxane (BAPTMDS)
- 1,3-Bis-[(diethylamino)-3-(propoxy)propan-2-ol]-1,1,3,3-tetramethyldisiloxane (BDEPPTMDS)
- 1,3,5,7-Tetrakis(3-polyethoxypropyl)-1,3,5,7-tetramethyltetracyclosiloxane methoxy terminated (TPEGTMCTS)
- 3-(Polyethoxypropyl)1,1,1,3,5,5,5-heptamethyltrisiloxane hydroxyl-terminated (PEGHMTS)
- 1,3-Bis(3-glycidyloxypropyl)-1,1,3,3-tetramethyldisiloxane (BGPTMDS)
- 1,3-Bis(3-polyethoxypropyl)-1,1,3,3-tetramethyldisiloxane methoxy-terminated (BPEGTMDS)

Due to their physiological neutrality, organosilicons are generally considered as environmentally friendly compounds. However, since low-molecular silanes and siloxanes may be hazardous for human health (under certain conditions they can overcome skin barriers and biological membranes), the adequate safety measures must be applied during its maintenance (i.e. protective mask, protective cloth and gloves, eye and face protection)^30^.

### Sample impregnation

Waterlogged wood samples of approximately 20 × 20 × 10 mm (radial × tangential × longitudinal direction) were dehydrated with 96% ethanol for 4 weeks and subsequently treated with 50% organosilicon in 96% ethanol (v/v) using the oscillating pressure method (−0.9 bar for 0.5 h and 10 bars for 6 h, repeated 6 times every 24 hours; between the cycles samples were left in the conservation solution for continuous soaking treatment). Then they were removed from the treatment solution and air-dried at ambient pressure and room temperature for 4 weeks. 6 replicates were used per each treatment and 6 untreated samples were used as a control.

### Effectiveness of the treatment

The effectiveness of the treatment was evaluated by calculating weight percent gain (WPG) of particular organosilicons according to the equation:

$$WPG=\frac{{W}_{1}-{W}_{0}}{{W}_{0}}\times 100$$

where W_0_ is the estimated oven-dry mass of the sample before treatment and W_1_ is the oven-dry mass of the treated sample.

Degraded waterlogged wood samples cannot be oven-dried before treatment because it would cause their irreversible shrinkage thus make them useless for the experiment. Therefore, for the above calculations, the estimated oven-dry mass of untreated samples calculated on the basis of water content in similar samples was used (5 replicates were used for the measurement).

### Infrared spectroscopy (FT-IR)

To evaluate the effectiveness of the treatment and confirm penetration of organosilicons into the wood structure, infrared spectra in the 4000–400 cm^−1^ region were recorded in KBr pellets on a Bruker ALPHA FT-IR spectrometer with a resolution of 4 cm^−1^. Internal parts of wood samples were air-dried, powdered and sieved, and the fraction with an average diameter less than 0.2 mm was retained for analysis. The wood sample concentration was of 2 mg in 200 mg KBr. For each sample, 5 recordings were performed and then the average spectrum was further used for the analysis. Because it is considered that the band from 1505 cm^−1^ (assigned to the stretching vibration of the aromatic ring in lignin) is not influenced by the treatment, all the spectra of the untreated and treated wood samples were normalized to it. Processing of the spectra was done by means of Grams 9.1 program (Thermo Fisher Scientific Inc.).

Impregnated and soaked/dried samples (tested for reversibility) were recorded on a Bruker Tensor 27 Fourier transform spectrometer with a resolution of 2 cm^−1^ equipped with a SPECAC Golden Gate single reflection diamond ATR (attenuated total reflection) unit. Measurements were carried out at 5 sites for each sample and the spectra were averaged using the OPUS Program.

### Dimensional stability

The dimensional stabilisation effect of the treatment was assessed by calculating volumetric shrinkage (S_v_) and anti-shrink efficiency coefficient (ASE_v_) according to standard equations:

$${S}_{v}=\frac{{V}_{0}-{V}_{1}}{{V}_{0}}\times 100$$

where V_0_ is the initial volume of the waterlogged sample and V_1_ is the final volume of the sample after drying,

$$AS{E}_{v}=\frac{{S}_{vu}-{S}_{vt}}{{S}_{vu}}\times 100$$

where S_vu_ and S_vt_ are the volumetric shrinkage of the untreated and treated sample, respectively.

The calculations were based on the pre- and post-treatment measurements of specimen dimensions in all three anatomical directions using a digital calliper (±0.01 mm).

### Scanning electron microscopy + EDX

Dry wood samples were imaged using the Quanta FEG 650 Scanning Electron Microscope (SEM) fitted with the Bruker X-Flash EDS detector (to acquire the X-ray maps for Si) at 15 and 25 kV beam energy. The cross-sections through the middle of the specimens were coated with a 15 nm layer of carbon using the high vacuum coating system (Leica EM ACE600) and analysed.

### Moisture content

Moisture content (MC) was determined using the standard oven-drying method and calculated as a ratio between the mass of water to the mass of a dry wood sample.

### Contact angle

Static water contact angle (WCA) measurements were performed using a Krüss GmbH DSA 100 Expert Drop Shape Analyser equipped with a software-controlled (DAS4 2.0): x, y, z-axis table, quadruple automatic dosing unit with zoom, focus, and illumination adjustment, and a camera of 780 × 580 pix resolution. Drop profile was extracted using a circle fitting model. Presented data are arithmetic means of 5 drops (5 µL volume) per sample.

### Reversibility of the treatment

In order to investigate the potential reversibility of the treatment, the air-dried and cured treated samples were soaked for two weeks in 50% ethanol solution (v/v) replaced for a fresh portion every 24 hours. FT-IR ATR measurements were performed before and after elution to assess the presence of particular organosilicons on the sample surface. The degree of an impregnate leaching was evaluated based on weight analysis of samples before soaking (control mass) and next immediately after 14 days of soaking, then after 2, 24, 48, 72, 168, and 336 hours of air drying at room temperature (c.a. 25 °C).

## Supplementary information

Supplementary information.

## Data Availability

The datasets generated during the current study are available from the corresponding author on reasonable request.
