# Supplementary material for: Organosilicons of different molecular size and chemical structure as consolidants for waterlogged archaeological wood – a new reversible and retreatable method
Source: Sci Rep. 2020 Feb 10;10:2188. doi: 10.1038/s41598-020-59240-8 (PMC7010770; doi:10.1038/s41598-020-59240-8)
Supplement: Supplementary file 1 — Supplementary information. [file 41598_2020_59240_MOESM1_ESM.pdf]

FT-IR analysis

Table S1. Band assignments and position for the silane treated wood samples [53, 54, 55, 56, 57, 58, 59, 60,61]

Bands assignment	Bands position						
	EA	MTMS	OTMS	MPTMS	TCPTMS	APTES	AEAPTMS
stretching vibration of OH groups in wood components<br>*Si-OH groups in silanols hydrogen bonded to molecular water*	3412	3415	3419	3418	3421	3424	3421
symmetric stretching vibration of C-H bonds in CH_3 groups in wood and *silanes*	2966	2975	2962	2963	2976	2962	2962
asymmetric stretching vibration of C-H bonds in CH_3 groups in wood and *silanes*	2936	2933	2925	2930	2938	2931	2932
symmetric stretching vibration of C-H bonds in CH_2 groups in wood and *silanes*	2878	2884	2884	2885	2887	2876	2880
asymmetric stretching vibration of C-H bonds in CH_2 groups in wood and *silanes*	2842	2842	2854	2851	2842		2849
N=C=S stretching vibration					2154		
N=C=S stretching vibration					2072		
C=O stretching vibration of carbonyl, carboxyl and acetyl groups	1738	1741	1740	1738	1737	1739	1740
conjugated C–O in quinines coupled with C=O stretching of various groups<br>*N-H bending vibration*	1663	1661	1656	1661	1663	1645	1653
absorbed O–H stretching	1634	1634	1631	1636	1631		
C=C stretching of aromatic skeletal (lignin)	1593	1596	1594	1595	1594	1594	1588
C-N stretching vibration in secondary amines						1563	1568
C=C stretching of aromatic skeletal (lignin)	1508	1510	1509	1510	1509	1501	1502
C–H deformation in lignin and carbohydrates<br>*C–H deformation in -CH_2-CH_3*	1461	1463	1463	1461	1461	1464	1470
C–H deformation in lignin and carbohydrates	1421	1421	1421	1421	1420	1428	1418

C–H asymmetric deformation in Si-R		1392				1385	1384
C–H deformation in cellulose and hemicellulose<br>*C-H asymmetric deformation of -CH_2 groups*	1367	1366	1375	1373	1378		1359
C–H vibration in cellulose and C_l–O vibration in syringyl derivatives – condensed structures in lignin<br>*CH_2 deformation vibration and C-N stretching vibration in primary amines*	1328	1331	1330	1340	1347	1347<br>1331	1323
-CH_2 groups twisting		1308	1301	1308	1309	1307	
C–O stretching in lignin<br>*symmetric C–H deformation in Si-CH_3*	1269	1271	1270	1265	1263	1268	1273
-CH_2 groups twisting				1238	1237		
C−O−C stretching mode of the pyranose ring	1221	1226	1219	1221	1221	1229	1228
C–O–C stretching vibration in cellulose and hemicelluloses	1192	1203			1193	1200	1203
asymmetric stretching vibration of Si-O-C		1169	1186	1187			
C–O–C stretching vibration in cellulose and hemicelluloses<br>*C-N stretching vibration*	1158					**1154**	**1153**
C–O stretching<br>*Si-O-C asymmetric stretching vibration*	1125	1129	1128	1129	1128	1129	1126
glucose ring stretching vibration<br>*asymmetric stretching vibration of Si-O-Si*	1087	1076	1081	1099	1096	1095	1094
C−O stretching vibrations in cellulose and hemicelluloses	1053						
C−O ester stretching vibrations in methoxyl and β–O–4 linkages in lignin<br>*Si-O-C asymmetric stretching vibration*	1029	1028	1026	1033	1030	1029	1029
Si-O-C stretching vibration				1004	1002	1000	1013
Si-O in-plane stretching vibration of the silanol Si-OH groups		958	955	953	957	952	
Si-O stretching vibration in un-condensed silanol (-SiOH groups)		908	920	918	906	915	921

Si-C symmetric stretching vibration		851		861	851	859	
Si-C symmetric stretching vibration			837			822	821
Si-O-Si bonds stretching vibration		813		807	805	789	784
-Si-C rocking in -SiCH$_3$		772	774	768	766	751	750
Si-O-Si bonds symmetric stretching vibration			690	692	695	694	695
O-Si-O deformation vibration		447	466	474	487	488	466

Table S2. Band assignments and position for the siloxane treated wood samples [53, 55, 56, 57, 60, 61, 62, 63]

Bands assignment	Bands position						
	EA	BAPTMDS	BDEPPTMDS	TPEGTMCTS	PEGHMTS	BGPTMDS	BPEGTMDS
stretching vibration of OH groups in wood components	3412	3373	3428	3436	3416	3412	3424
symmetric stretching vibration of C-H bonds in CH$_3$ groups in wood and *siloxanes*	2966	2957	2968	2957	2957	2955	2953
asymmetric stretching vibration of C-H bonds in CH$_3$ groups in wood and *siloxanes*	2936	2925	2931	2928	2924	2928	2929
C-H stretching vibration in siloxane		2896	2902		2905		
symmetric stretching vibration of C-H bonds in CH$_2$ groups in wood and *siloxanes*	2878	2870	2869	2869	2866	2860	2866
asymmetric stretching vibration of C-H bonds in CH$_2$ groups in wood and *siloxanes*	2842						
C-H stretching vibration in siloxane		2795	2802	2813	2798	2976	2811
C-H stretching vibration in siloxane			2754				2774
C-H stretching vibration in siloxane			2723	2739	2737		

C-H stretching vibration in siloxane				2697	2697		
C=O stretching vibration of carbonyl, carboxyl and acetyl groups	1738	1744	1738	1737	1736	1740	1737
conjugated C–O in quinines coupled with C=O stretching of various groups	1663	1666	1664	1650	1662	1665	1665
absorbed O–H stretching	1634	1637			1639	1637	1634
C=C stretching of aromatic skeletal (lignin)	1593	1590	1594	1592	1594	1593	1592
C-N stretching vibration in amines		1568	1551	1550		1551	1550
C=C stretching of aromatic skeletal (lignin)	1508	1507	1509	1511	1510	1509	1510
C–H deformation in lignin and carbohydrates	1461	1464	1464	1461	1460	1462	1460
C–H deformation in lignin and carbohydrates<br>*C-H deformation vibration in siloxane*	1421	1417	1416	1417	1416	1416	1416
C-N stretching vibration in amines		1384	1384	1390	1386	1372	1384
C–H deformation in cellulose and hemicellulose	1367		1374			1371	
CH_2 deformation vibration				1353	1352		1352
C–H vibration in cellulose and C_l–O vibration in syringyl derivatives – condensed structures in lignin<br>*CH_2 deformation vibration in R*<br>*C-N stretching vibration in primary amines*	1328	1336	1334	1320	1322	1331	1323
-CH_2 groups twisting		1301	1295	1299	1299	1313	1298
C–O stretching in lignin	1269						
symmetric C–H deformation in Si-CH_3		1256	1254	1259	1257	1254	1254
C−O−C stretching mode of the pyranose ring	1221	1220	1218		1217	1219	1220

C–O–C stretching vibration in cellulose and hemicelluloses<br>*C-O-C stretching vibration*	1192		1201	1196	1192	1188	1197
asymmetric stretching vibration of Si-O-Si		1183	1181				
C–O–C stretching vibration in cellulose and hemicelluloses<br>*C-N stretching vibration*	1158	1156				1158	
C-N stretching vibration				1142	1146		1146
C–O stretching<br>*Si-O-Si asymmetric stretching vibration*	1125	1128	1123		1119		
Si-O-Si stretching vibration				1103		1104	1108
glucose ring stretching vibration<br>*asymmetric stretching vibration of Si-O-Si*	1087				1081		
C−O stretching vibrations in cellulose and hemicelluloses<br>*C−O stretching vibration*	1053		1062				
Si-O-Si asymmetric stretching vibration due to disiloxane structure		1042	1041		1041	1048	
C−O ester stretching vibrations in methoxyl and β–O–4 linkages in lignin<br>*C−O stretching vibration*	1029			1025			1036
Si-O-C stretching vibration		1003	987	987	987	984	987
Si-O in-plane stretching vibration		954	957	949	949	955	949
Si-O stretching vibration		892	932 / 900	908		897	
Si-C symmetric stretching vibration		840	840	846	842	840	841
Si-O-Si bonds stretching vibration		796	795	803	790	796	797
-Si-C rocking in -SiCH$_3$		771	776	758	755	778	775
Si-O-Si bonds symmetric stretching vibration		706	705	699	690	704	704

Si-O-Si bonds symmetric stretching vibration		680	679				
Si-O deformation vibration		469	465				

FT-IR ATR analysis

Table S3. Characteristic bands observed in FT-IR spectra of impregnated wood samples[64]; [a] beside the bands characteristic for a raw wood sample

Silane or siloxane compound	Characteristic FT-IR bands [cm^{-1}] and their assignment[a]
-	3343 (O-H); 2918, 2850 (methylene C-H); 1592 (C=C), 1506 (C=C), 1460, 1420, 1370 (methyl and methylene C-H); 1327 (methylene C-H, O-H); 1265, 1223 (C-O); 1123, 1031 (C-O-C).
APTES	3282 (overlapping N-H and O-H); 2926, 2902 (methyl and methylene C-H); 1563 (C-N), 1481, 1430 (methyl and methylene C-H); 1320 (methylene C-H, O-H); 1109 (Si-O-C); 1015 (Si-O-Si); 931, 812, 775, 750, 692 (overlapping methyl and methylene C-H, and N-H).
AEAPTES	3291 (overlapping N-H and O-H); 1571 (C-N); 1469 (methyl and methylene C-H); 1313 (methylene C-H, O-H); 1104 (Si-O-C); 1020 (S-O-Si); 926, 810, 773, 721, 693 (overlapping methyl and methylene C-H, and N-H).
MTMS	2967, 1270 (methyl C-H); 1028 (overlapping Si-O-C and Si-O-Si); 912, 778 (methyl C-H).
OTMS	2956, 2923, 2854 (methyl and methylene C-H); 1105, 1079, 1033 (overlapping Si-O-C and Si-O-Si); 772, 688 (methyl and methylene C-H).
BAPTMDS	3276 (overlapping N-H and O-H); 2953, 2923, 2895, 2857 (methyl and methylene C-H); 1636 (N-H); 1565 (C-N); 1442, 1402, 1327, 1250 (methyl and methylene C-H); 1055 (Si-O-Si), 837, 787, 703 (overlapping methyl and methylene C-H, and N-H).
MPTMS	1107, 1032 (overlapping Si-O-C and Si-O-Si); 914, 802, 691 (methyl and methylene C-H).

BDEPPTMDS	2968, 2930, 2871, 1266 (methyl and methylene C-H); 1108 (C-O-C); 1049 (Si-O-Si), 839, 770 (methyl and methylene C-H).
TPEGTMCTS	2868 (methylene C-H); 1455, 1350, 1259 (methyl C-H); 1094 (C-O-C); 1031 (Si-O-Si) 948, 908, 847, 801 (methyl and methylene C-H).
PEGHMTS	2954 (methyl C-H); 2869 (methylene C-H); 1349 (methylene C-H); 1254 (methyl C-H); 1038 (Si-O-Si and C-O-C); 951, 840, 755 (methyl and methylene C-H).
TCPTMS	2152 (S-C≡N); 1116, 1029 (overlapping Si-O-C and Si-O-Si); 910, 795, 692 (methyl and methylene C-H).
BGPTMDS	2952, 2931, 2864, 2800, 1252, (methyl and methylene C-H); 1098 (C-O-C); 1041 (Si-O-Si); 837, 777, 702, 617 (methyl and methylene C-H).
BPEGTMDS	2868 (methylene C-H); 1456, 1350, 1253(methyl and methylene C-H); 1103 (C-O-C); 1034 Si-O-Si), 950, 839, 780 (methyl and methylene C-H).

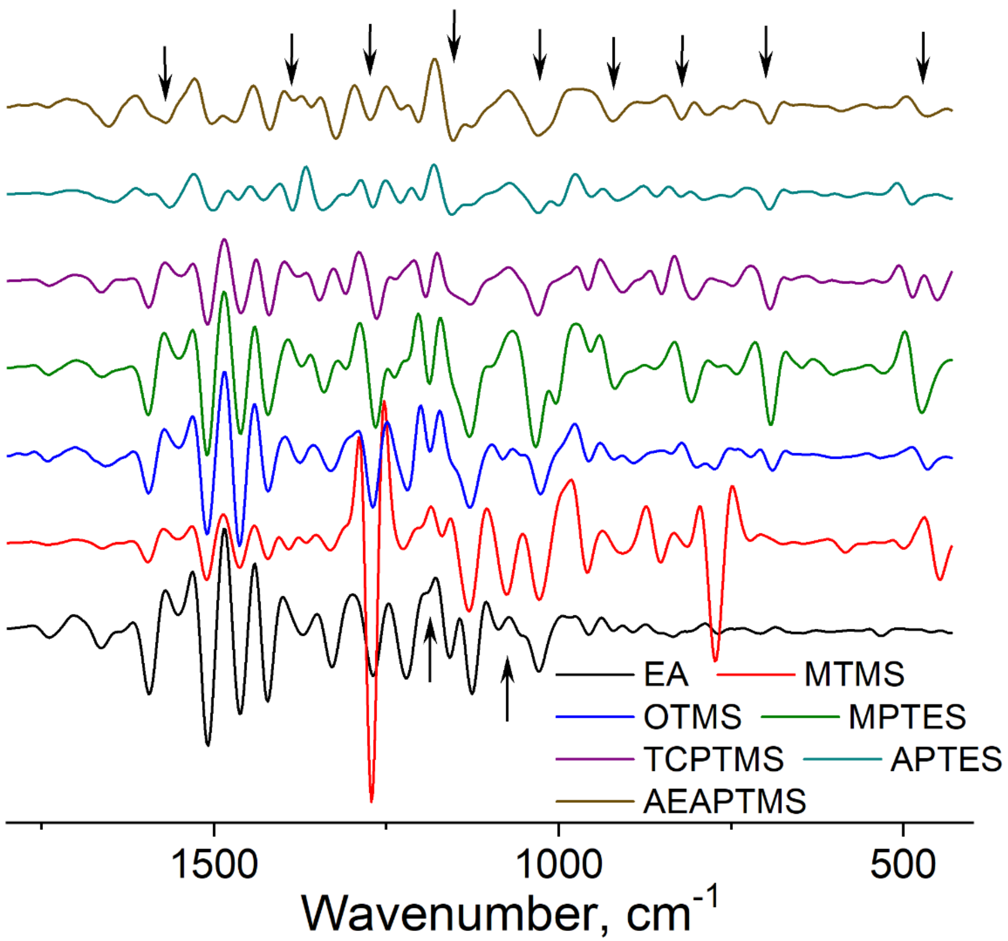

Fig. S1a. Second derivative spectra of silane- treated waterlogged elm samples, the main bands marked with arrows

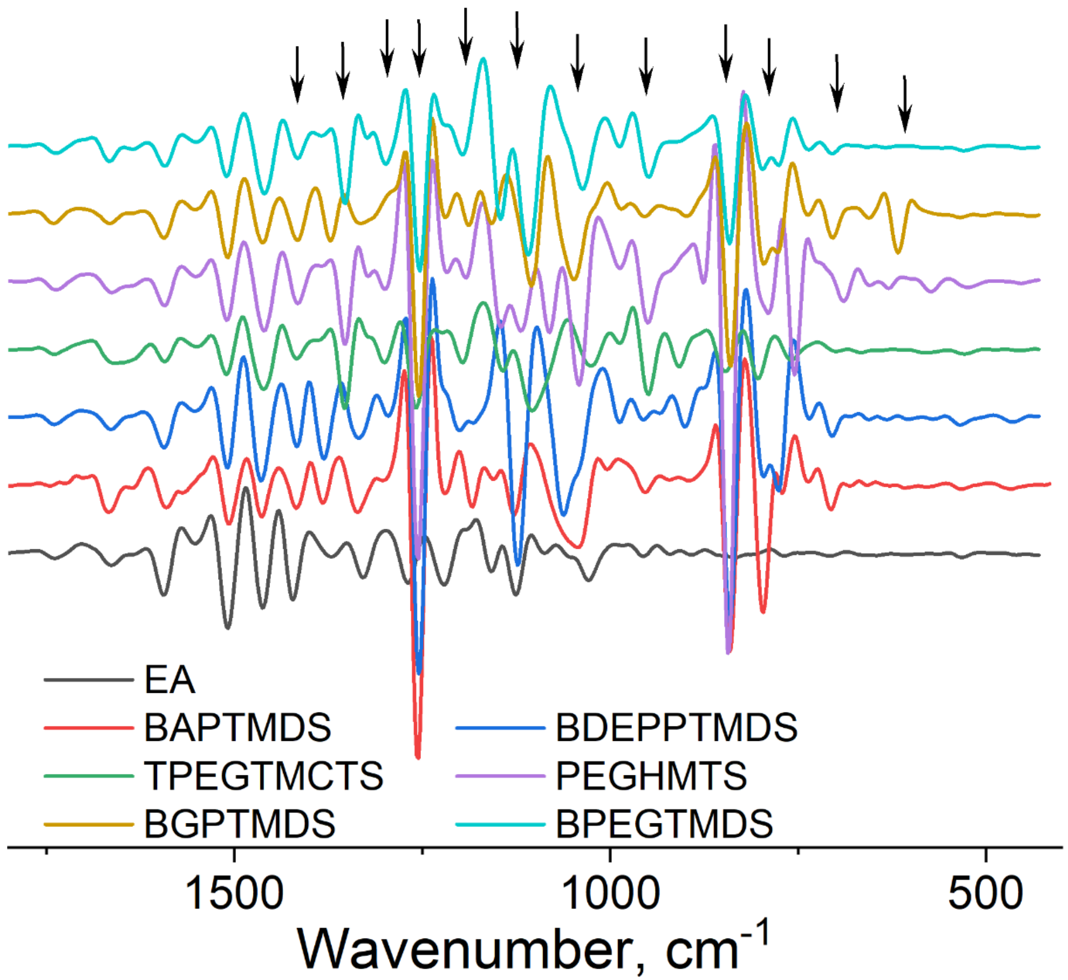

Fig. S1b. Second derivative spectra of siloxane-treated waterlogged elm samples, the main bands marked with arrows

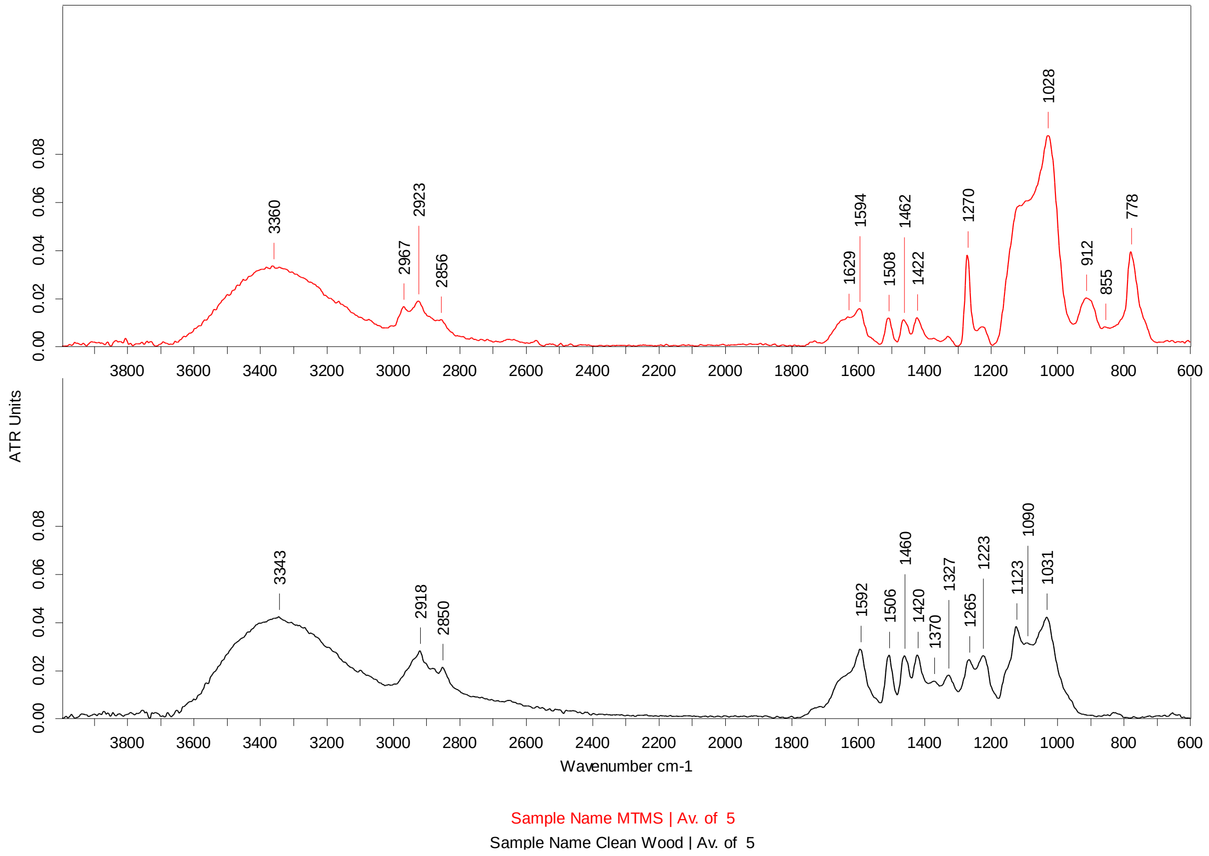

Fig. S2. Stacked FT-IR spectra of clean wood sample (black) and wood sample treated with MTMS (red)

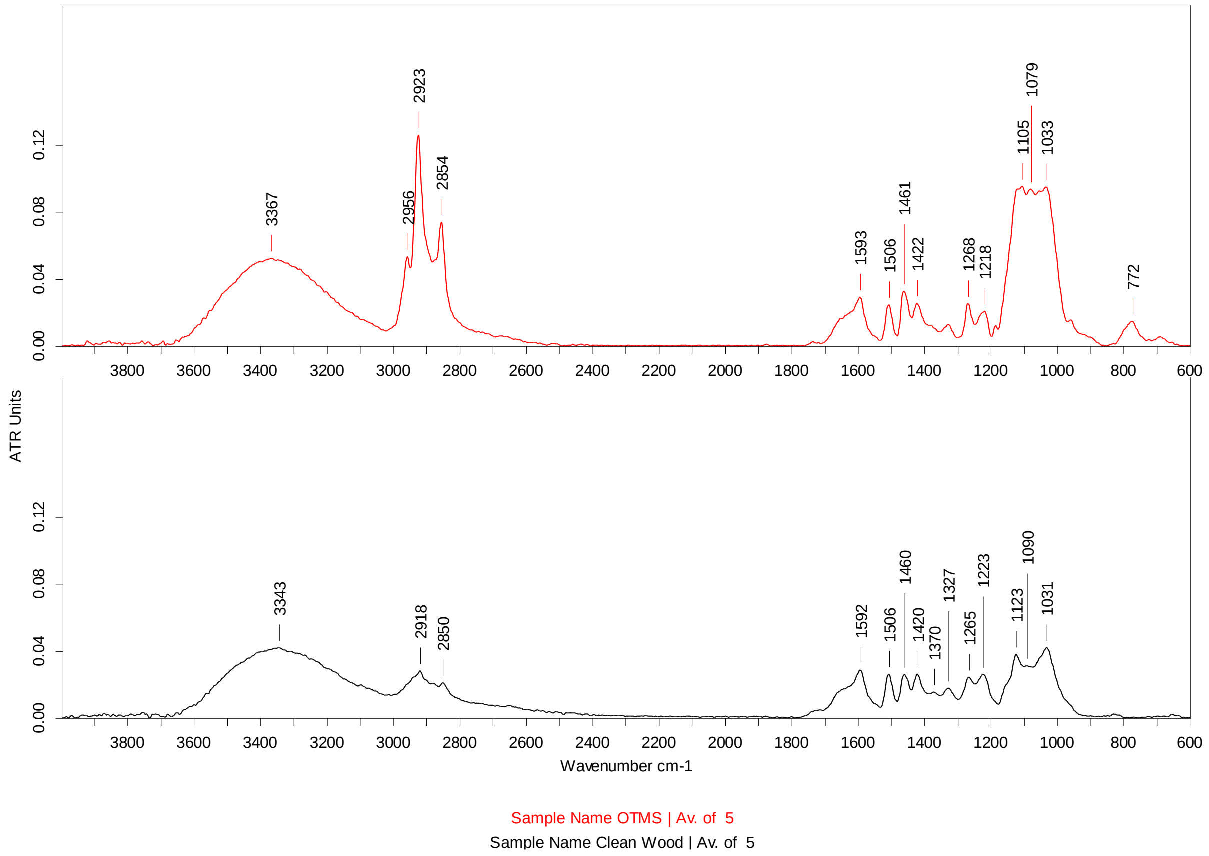

Fig. S3. Stacked FT-IR spectra of clean wood sample (black) and wood sample treated with OTMS (red)

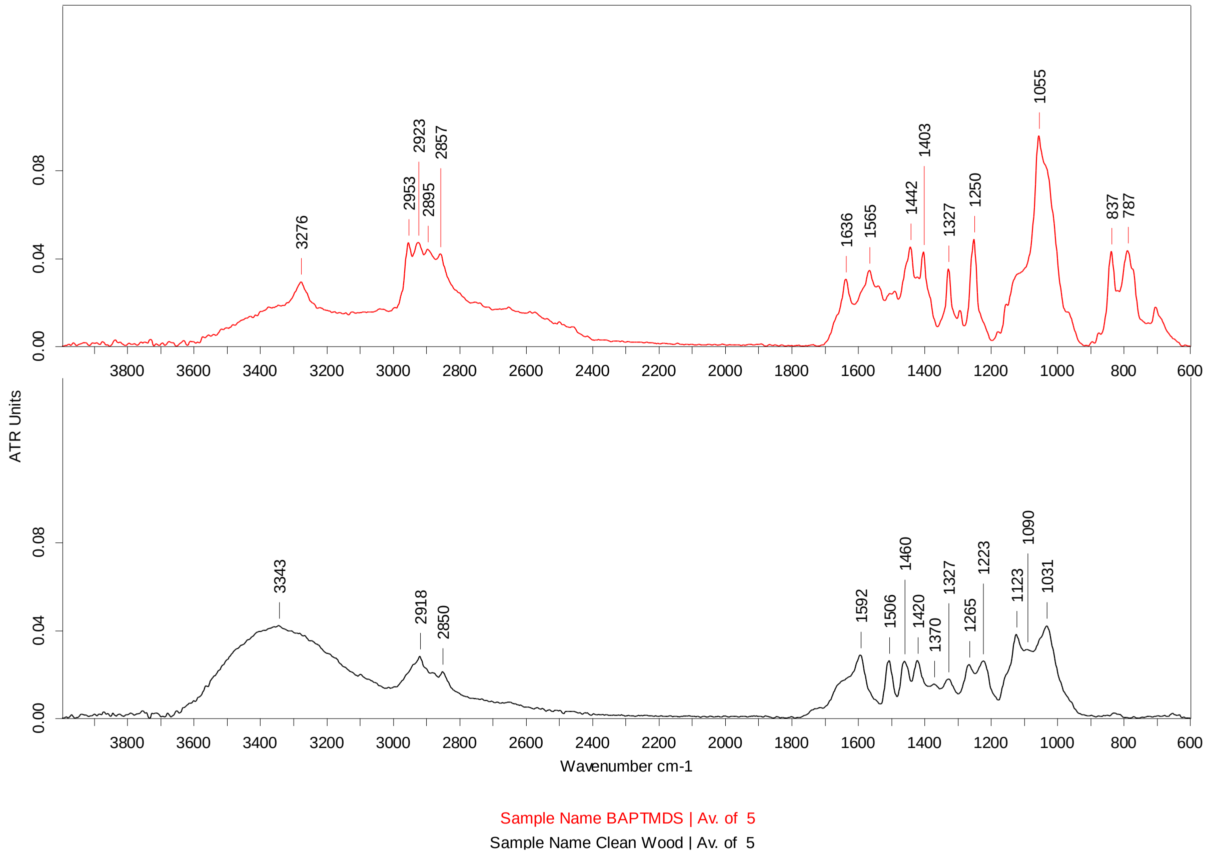

Fig. S4. Stacked FT-IR spectra of clean wood sample (black) and wood sample treated with BAPTMDS (red)

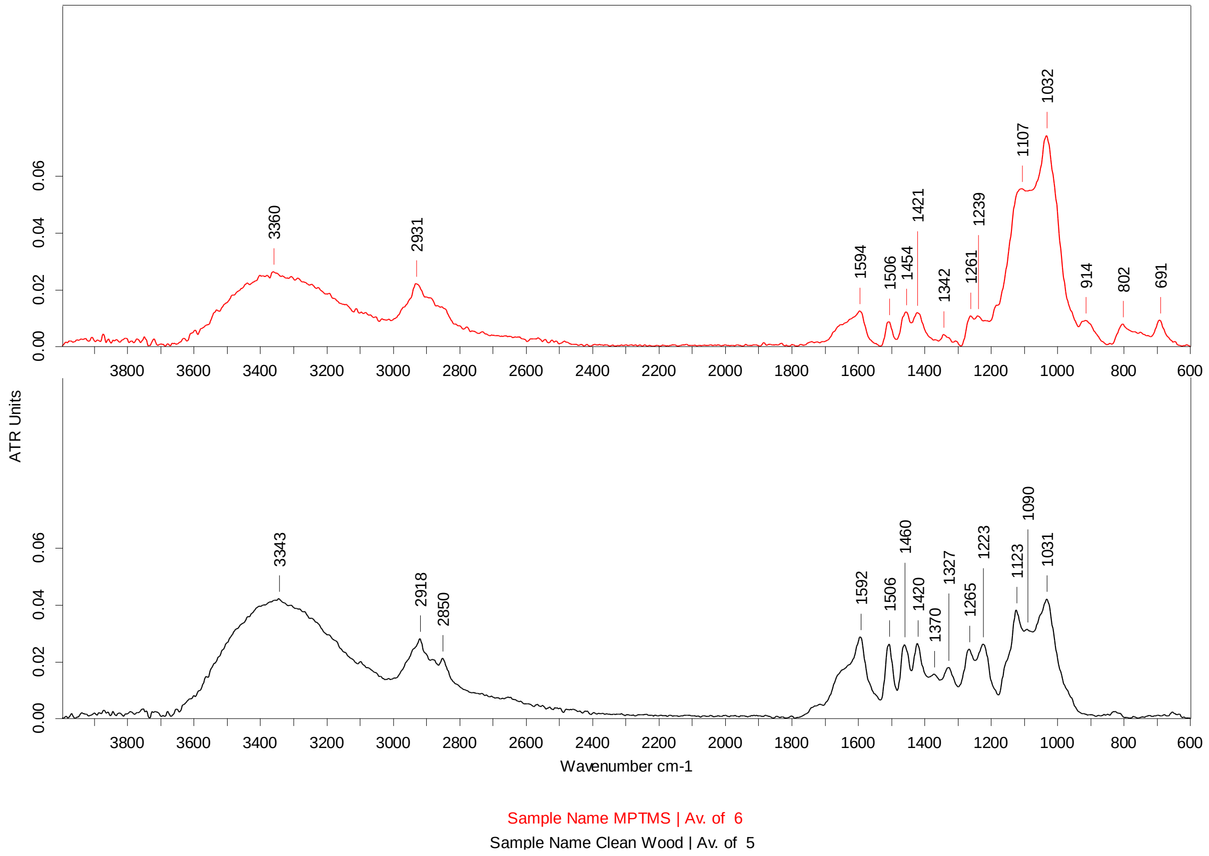

Fig. S5. Stacked FT-IR spectra of clean wood sample (black) and wood sample treated with MPTMS (red)

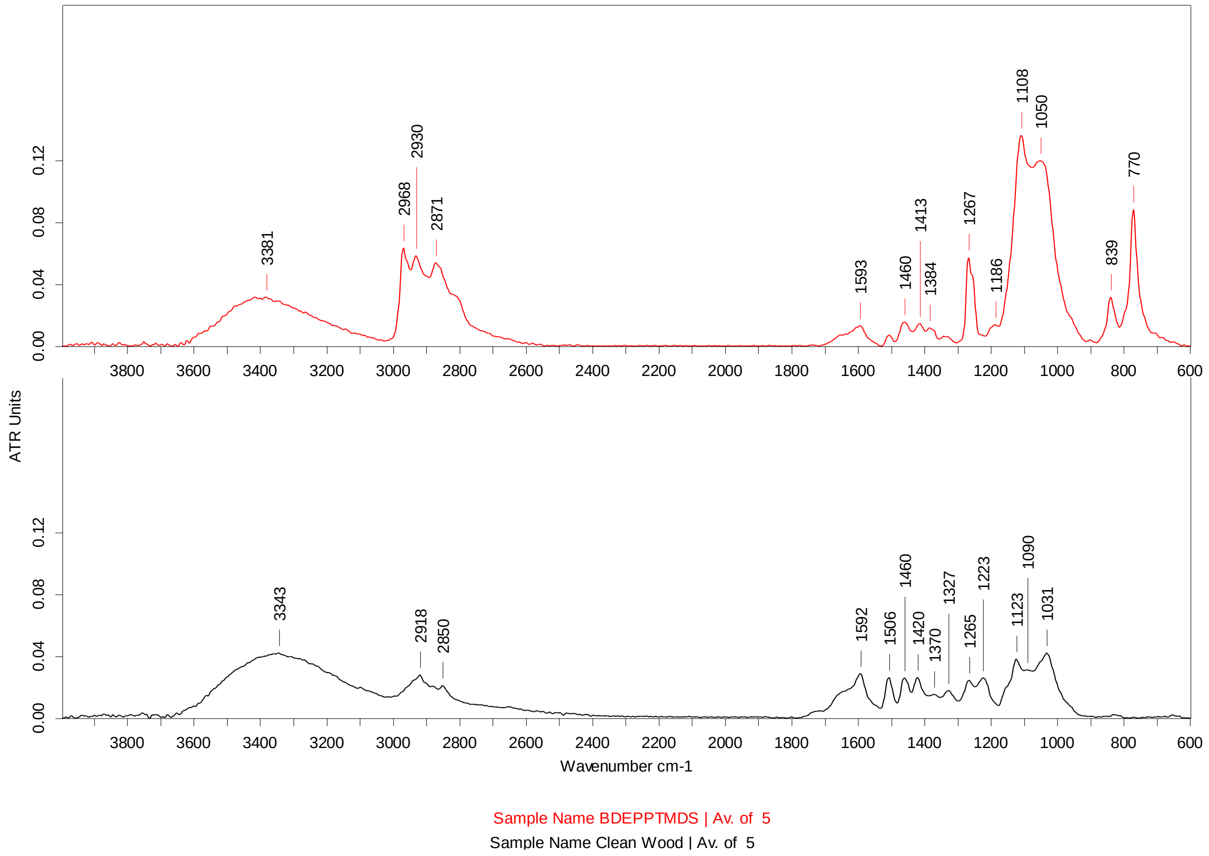

Fig. S6. Stacked FT-IR spectra of clean wood sample (black) and wood sample treated with BDEPPTMDS (red)

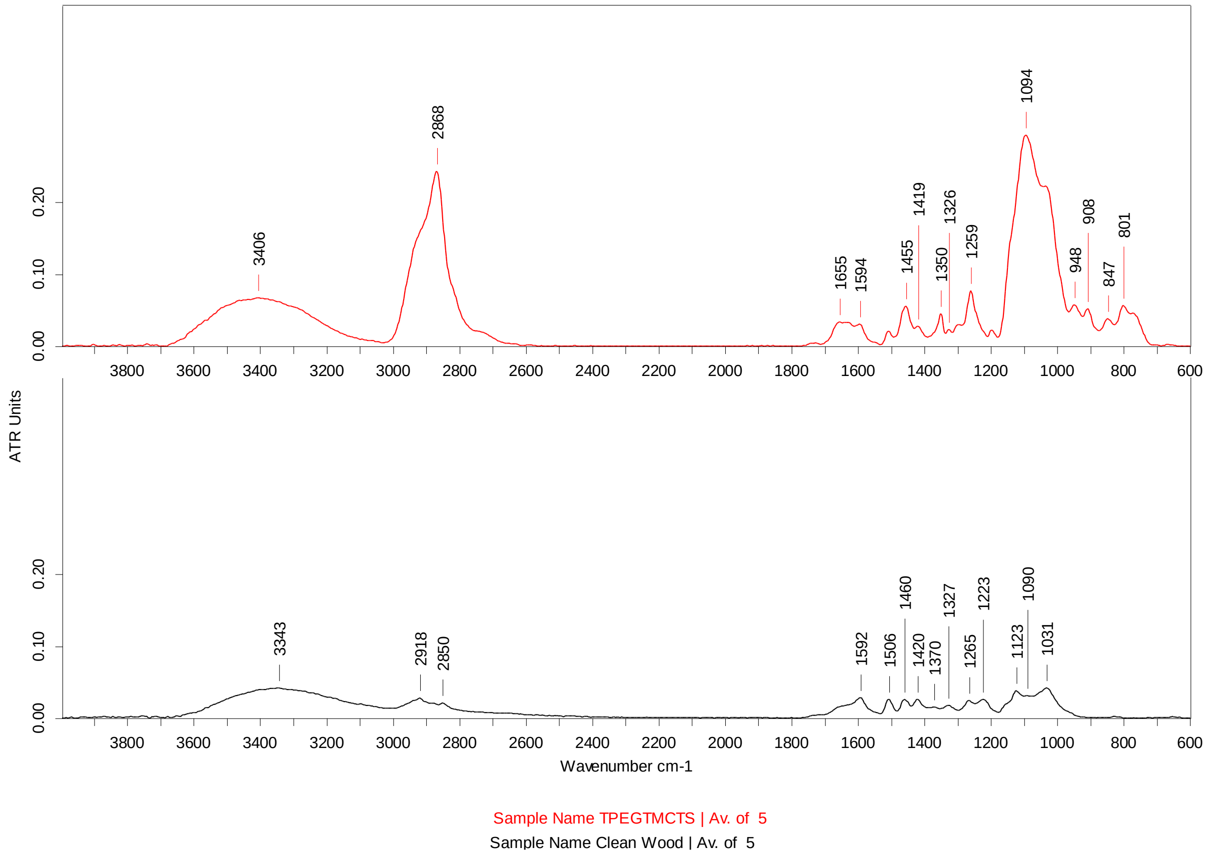

Fig. S7. Stacked FT-IR spectra of clean wood sample (black) and wood sample treated with TPEGTMCTS (red)

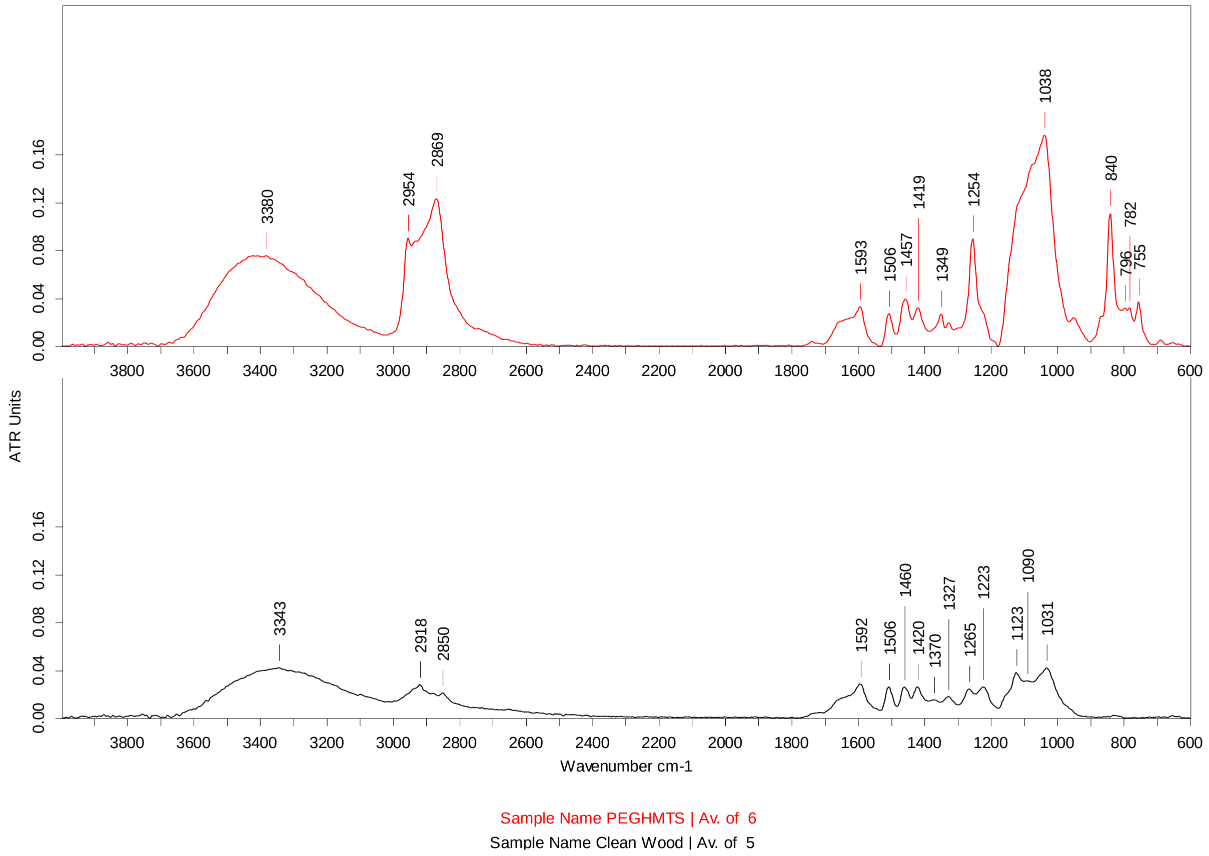

Fig. S8. Stacked FT-IR spectra of clean wood sample (black) and wood sample treated with PEGHMTS (red)

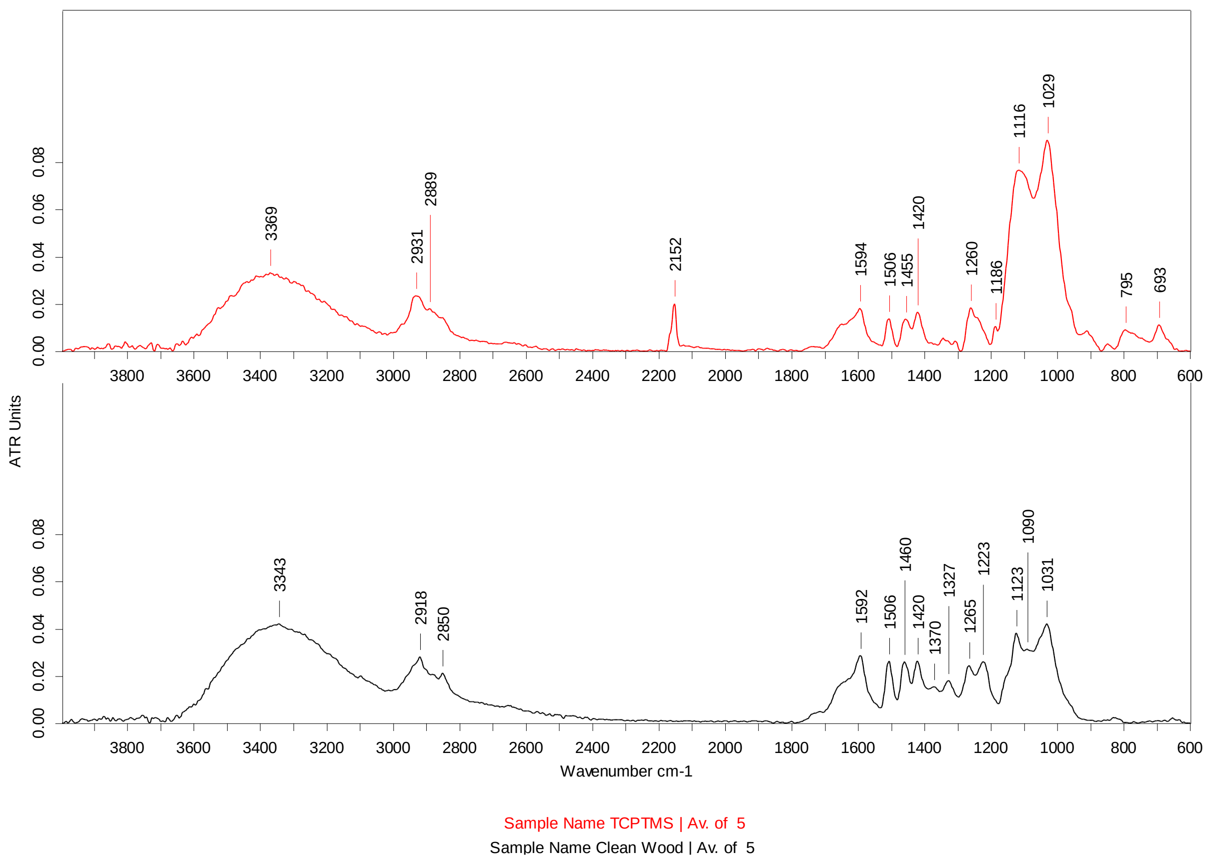

Fig. S9. Stacked FT-IR spectra of clean wood sample (black) and wood sample treated with TCPTMS (red)

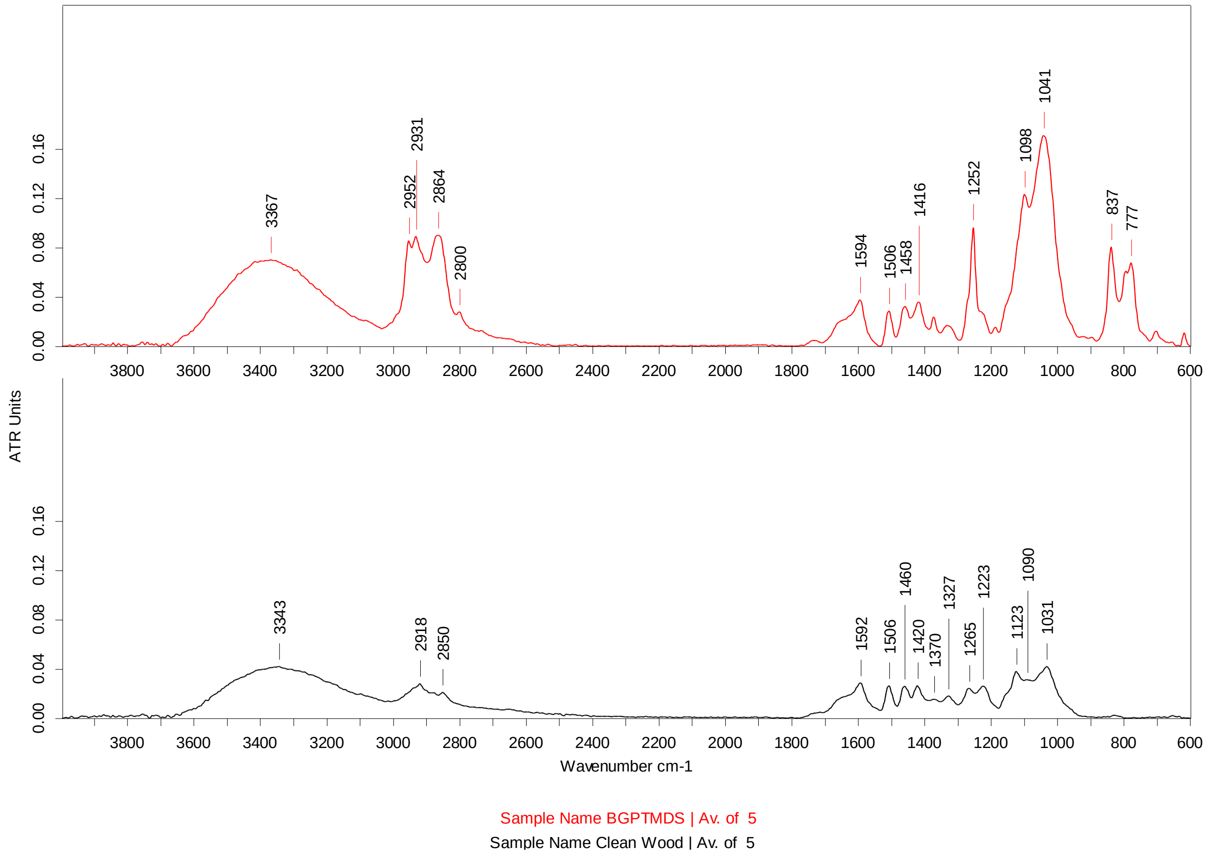

Fig. S10. Stacked FT-IR spectra of clean wood sample (black) and wood sample treated with BGPTMDS (red)

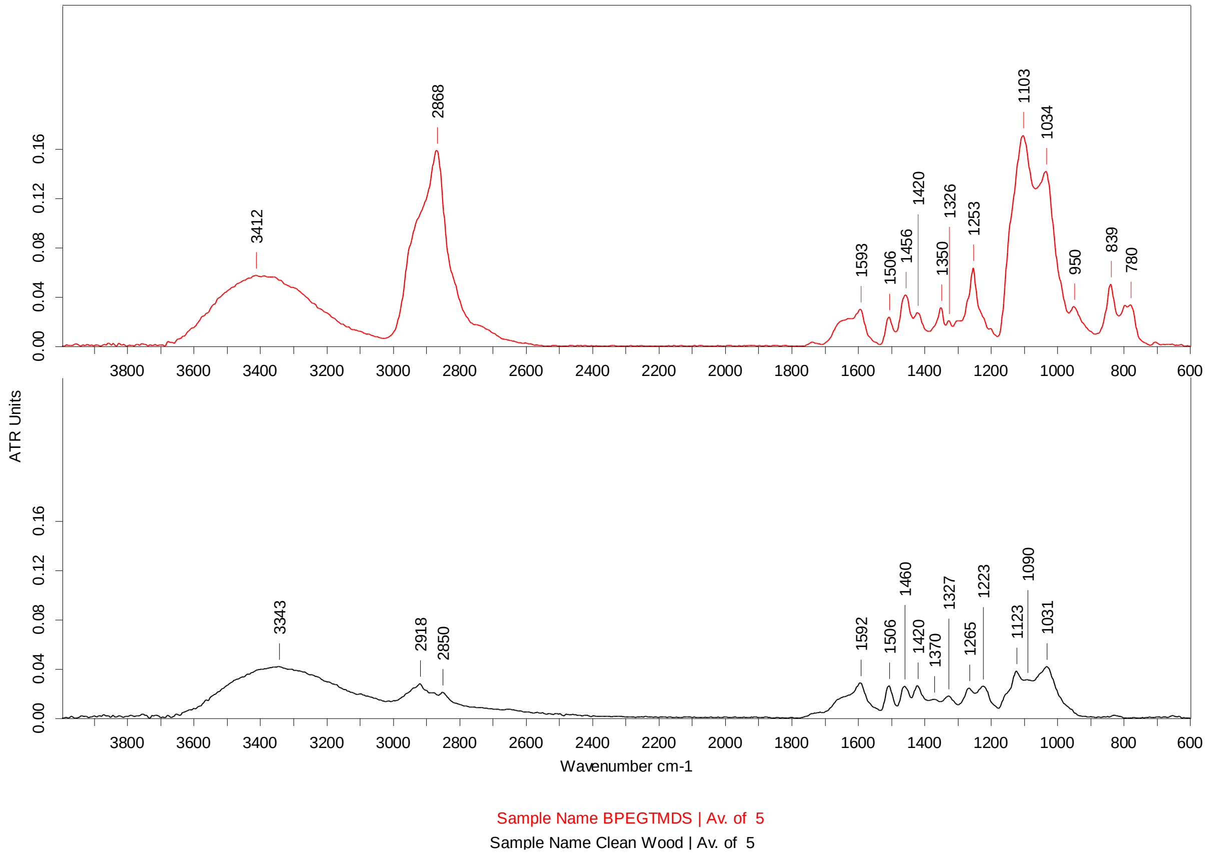

Fig. S11. Stacked FT-IR spectra of clean wood sample (black) and wood sample treated with BPEGTMDS (red)

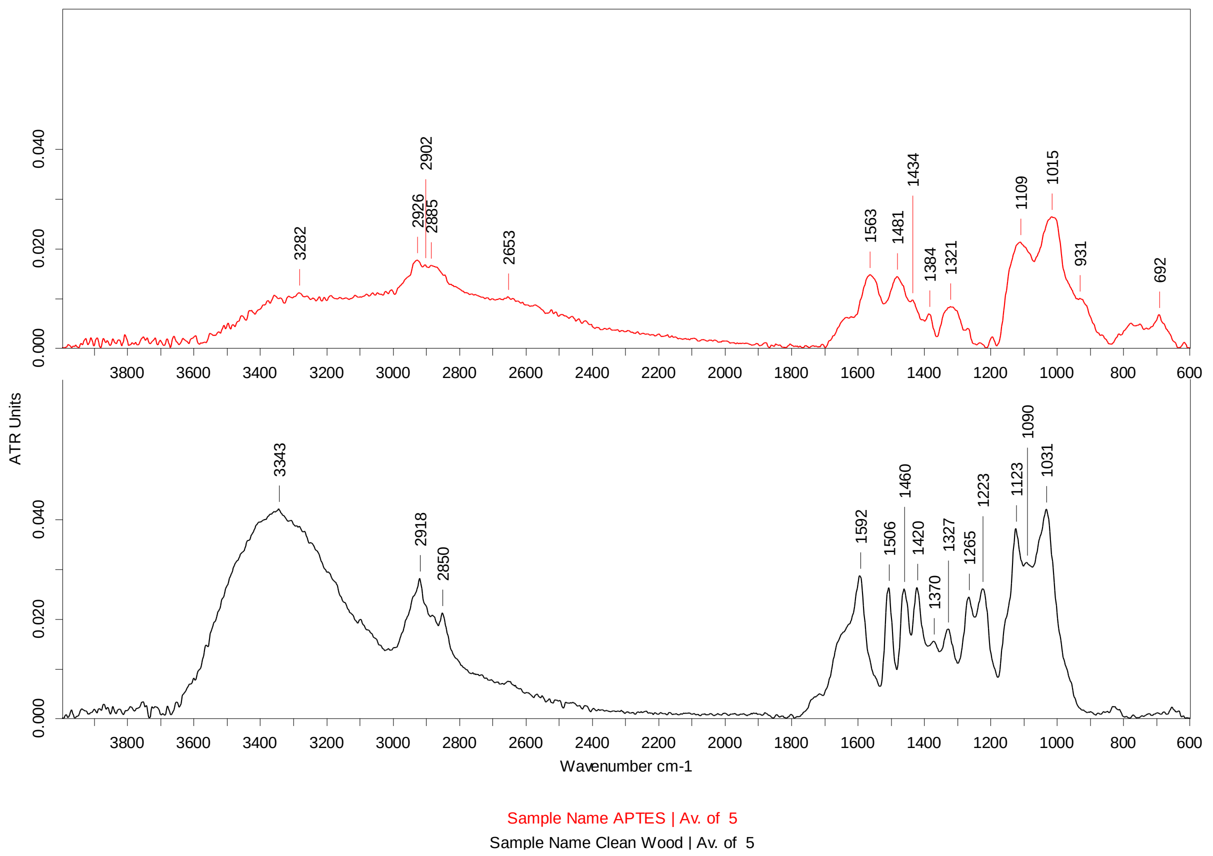

Fig. S12. Stacked FT-IR spectra of clean wood sample (black) and wood sample treated with APTES (red)

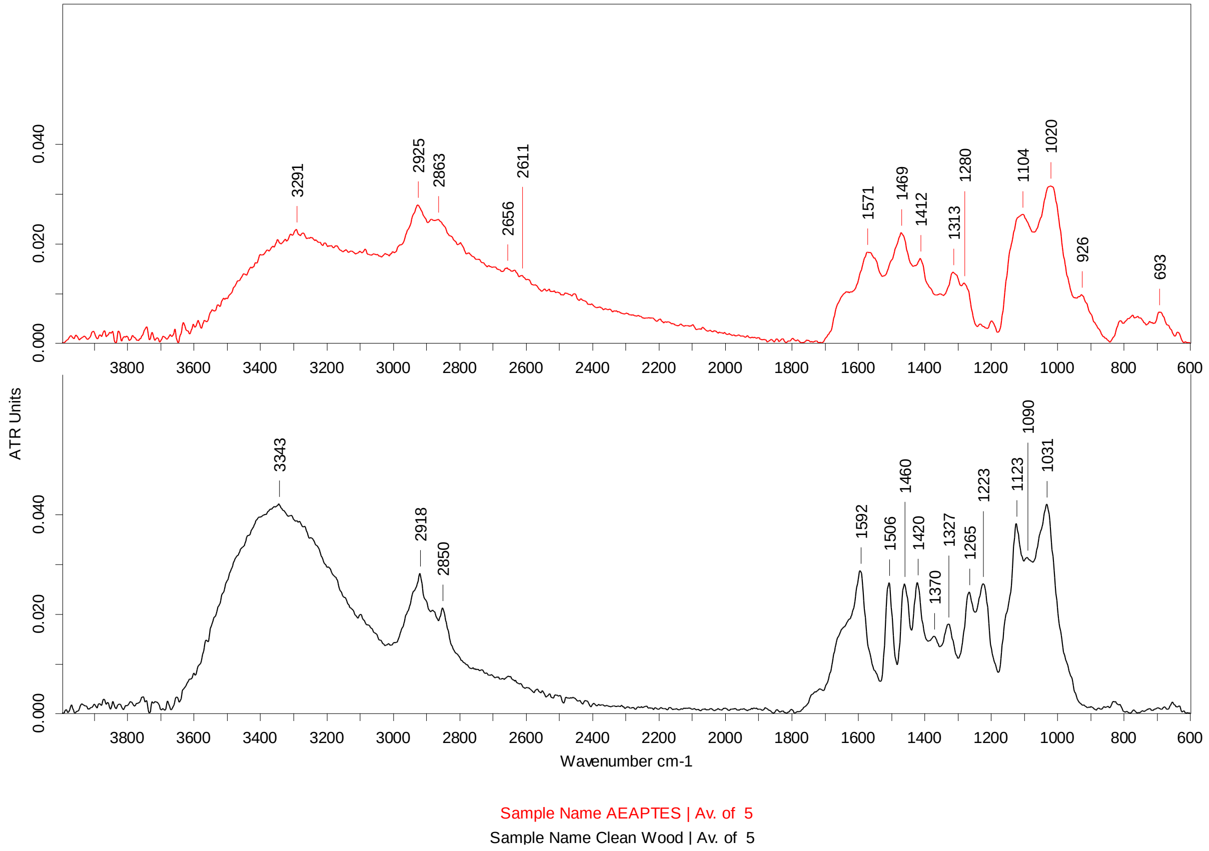

Fig. S13. Stacked FT-IR spectra of clean wood sample (black) and wood sample treated with AEAPTES (red)

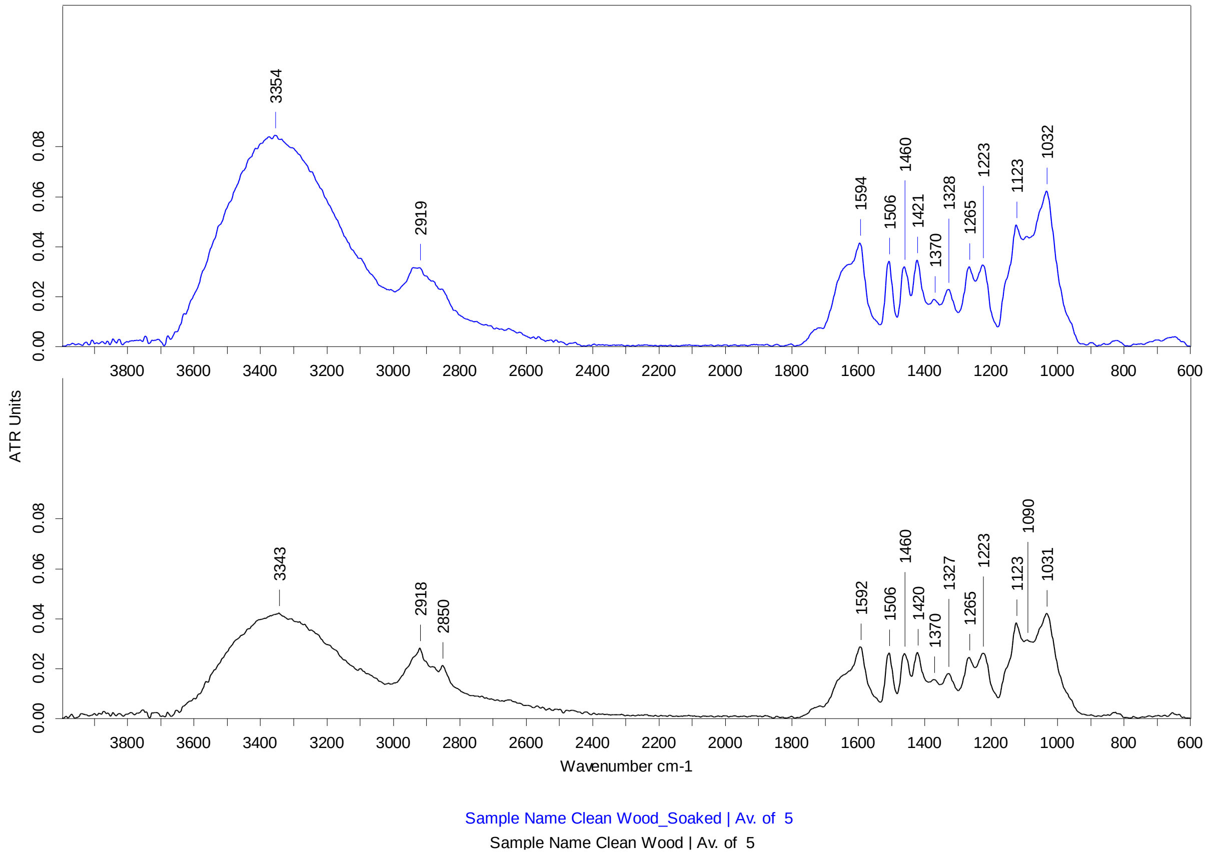

Fig. S14. Stacked FT-IR spectra of clean wood sample before (black) and after soaking (blue)

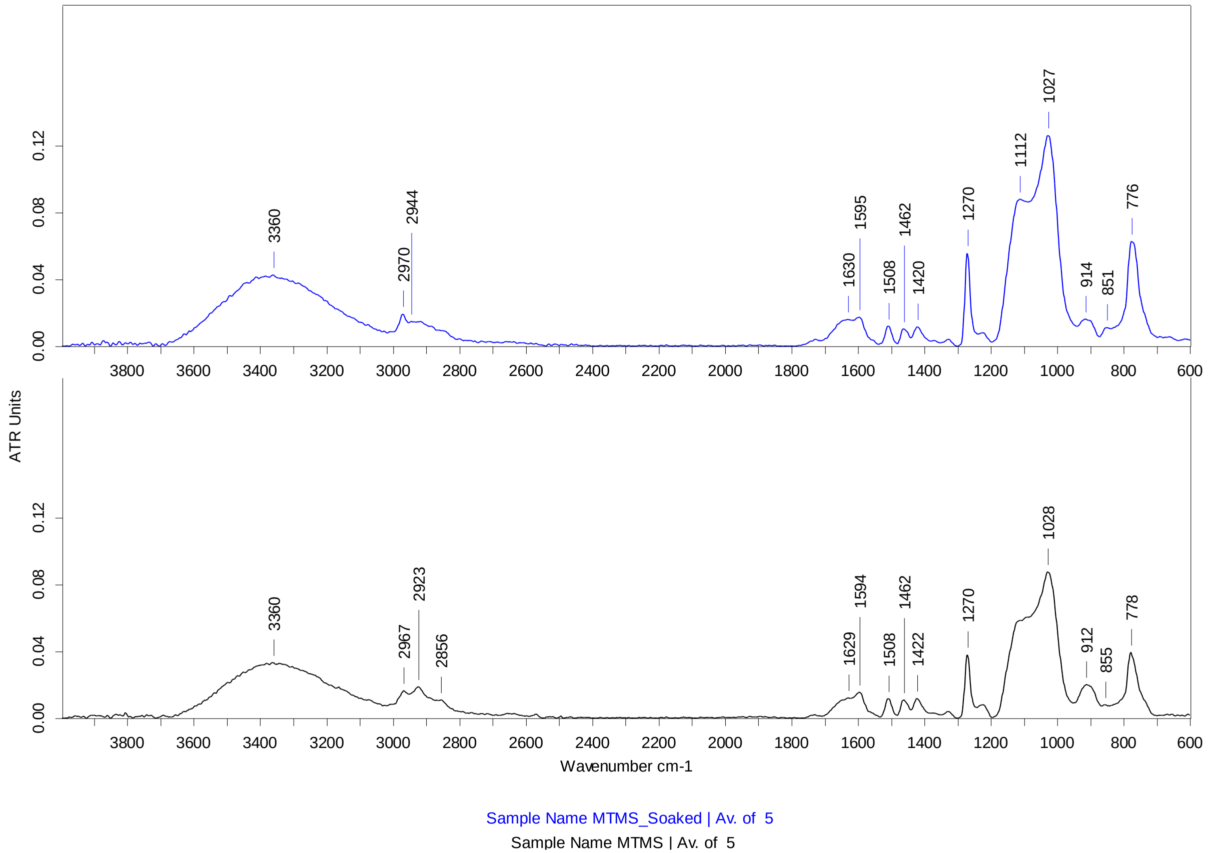

Fig. S15. Stacked FT-IR spectra of wood sample treated with MTMS before (black) and after soaking (blue)

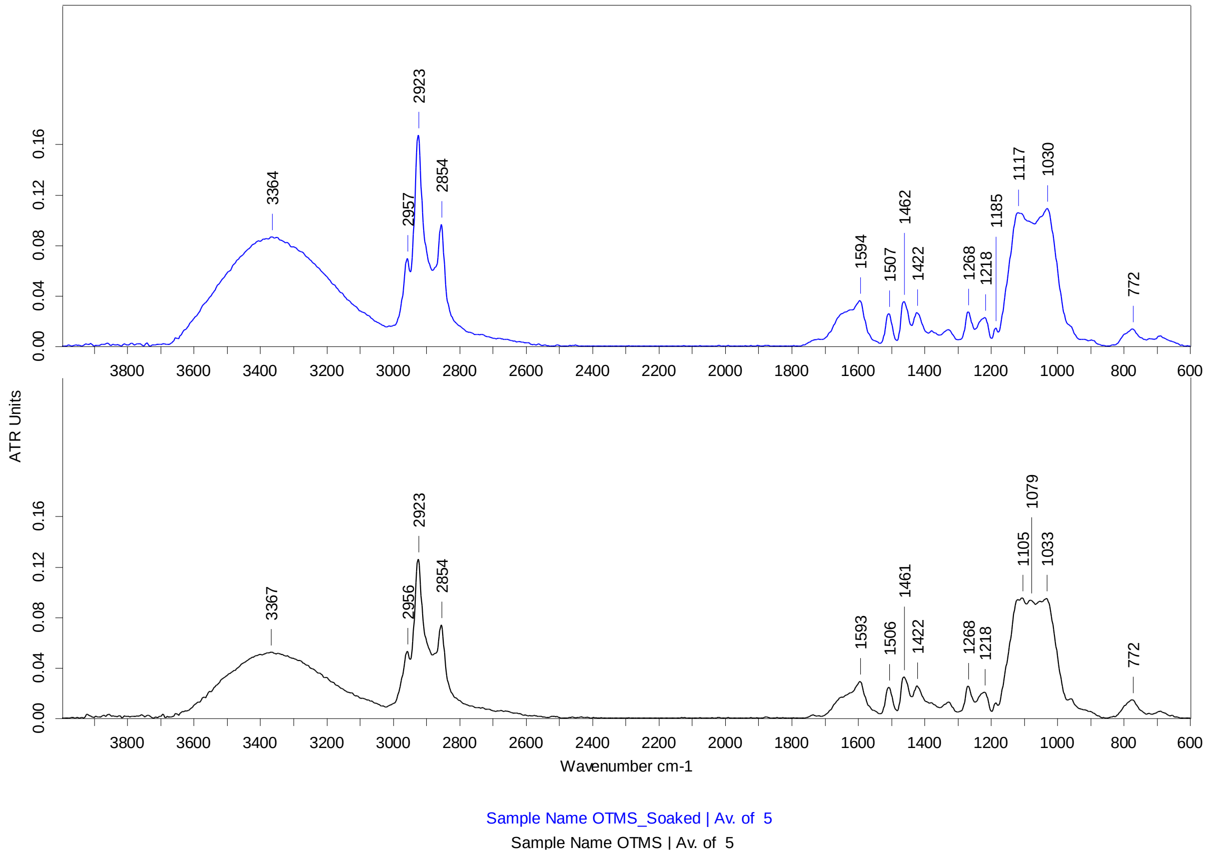

Fig. S16. Stacked FT-IR spectra of wood sample treated with OTMS before (black) and after soaking (blue)

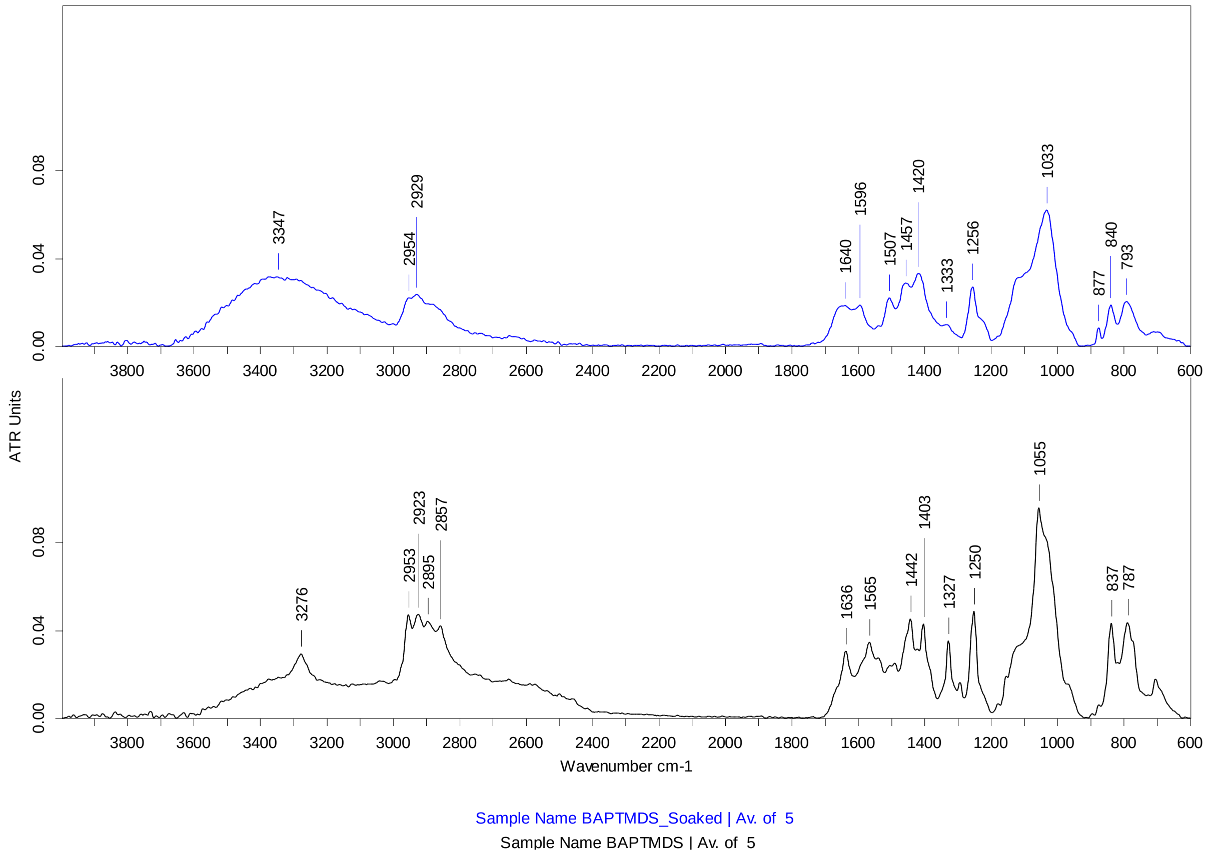

Fig. S17. Stacked FT-IR spectra of wood sample treated with BAPTMDS before (black) and after soaking (blue)

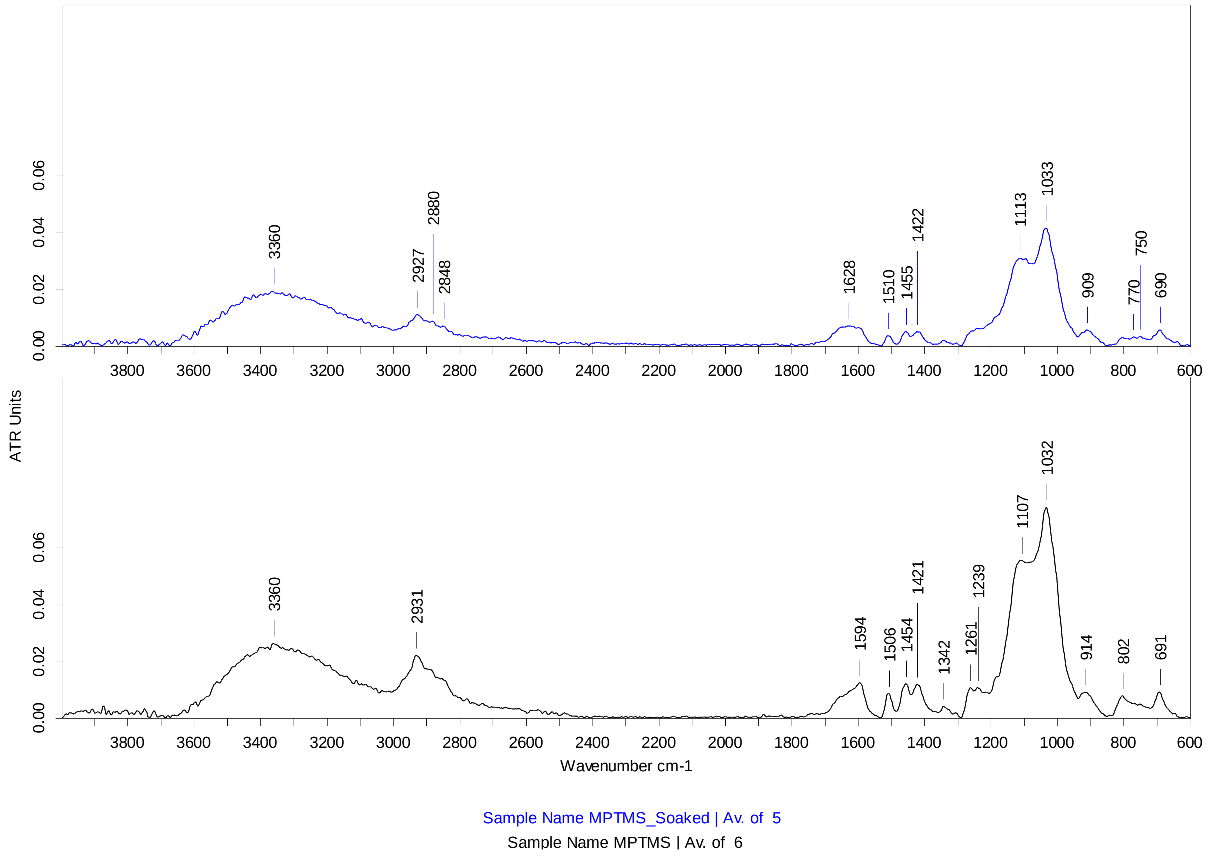

Fig. S18. Stacked FT-IR spectra of wood sample treated with MPTMS before (black) and after soaking (blue)

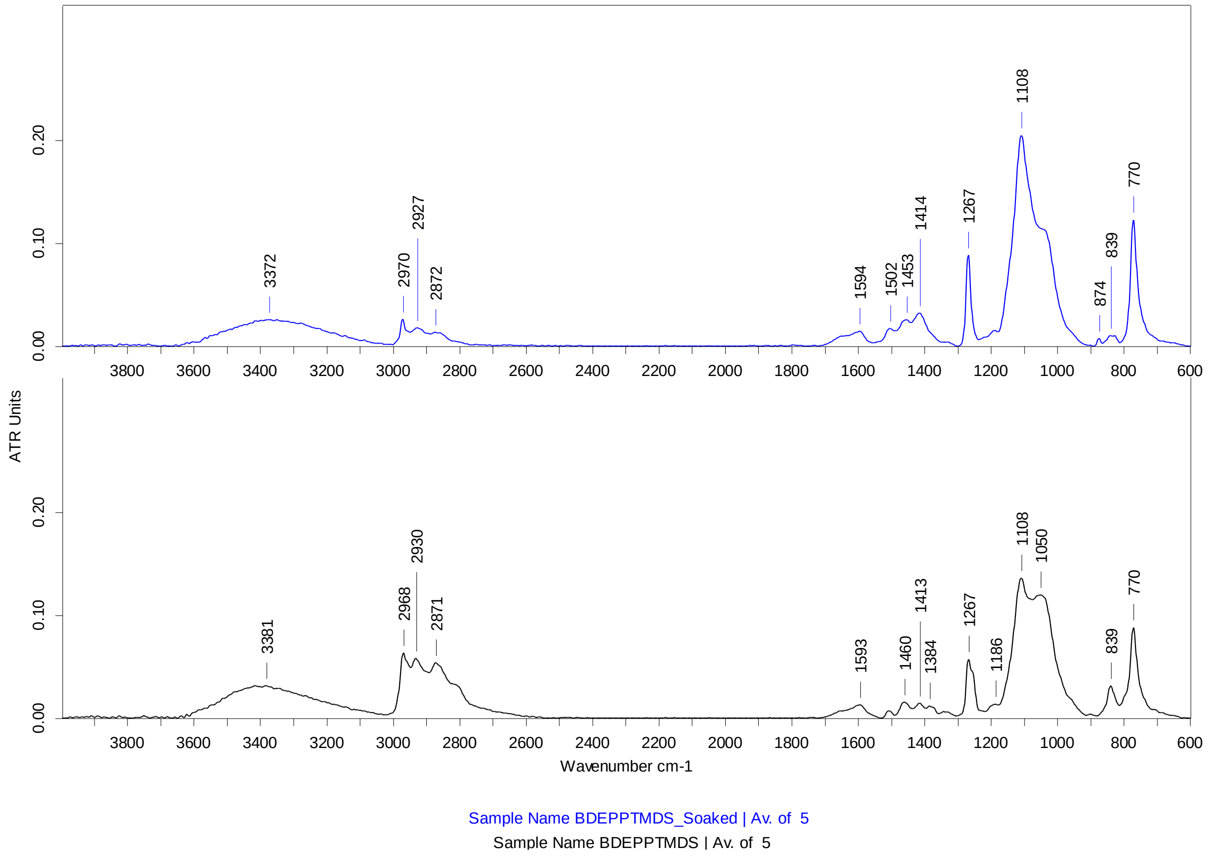

Fig. S19. Stacked FT-IR spectra of wood sample treated with BDEPPTMDS before (black) and after soaking (blue)

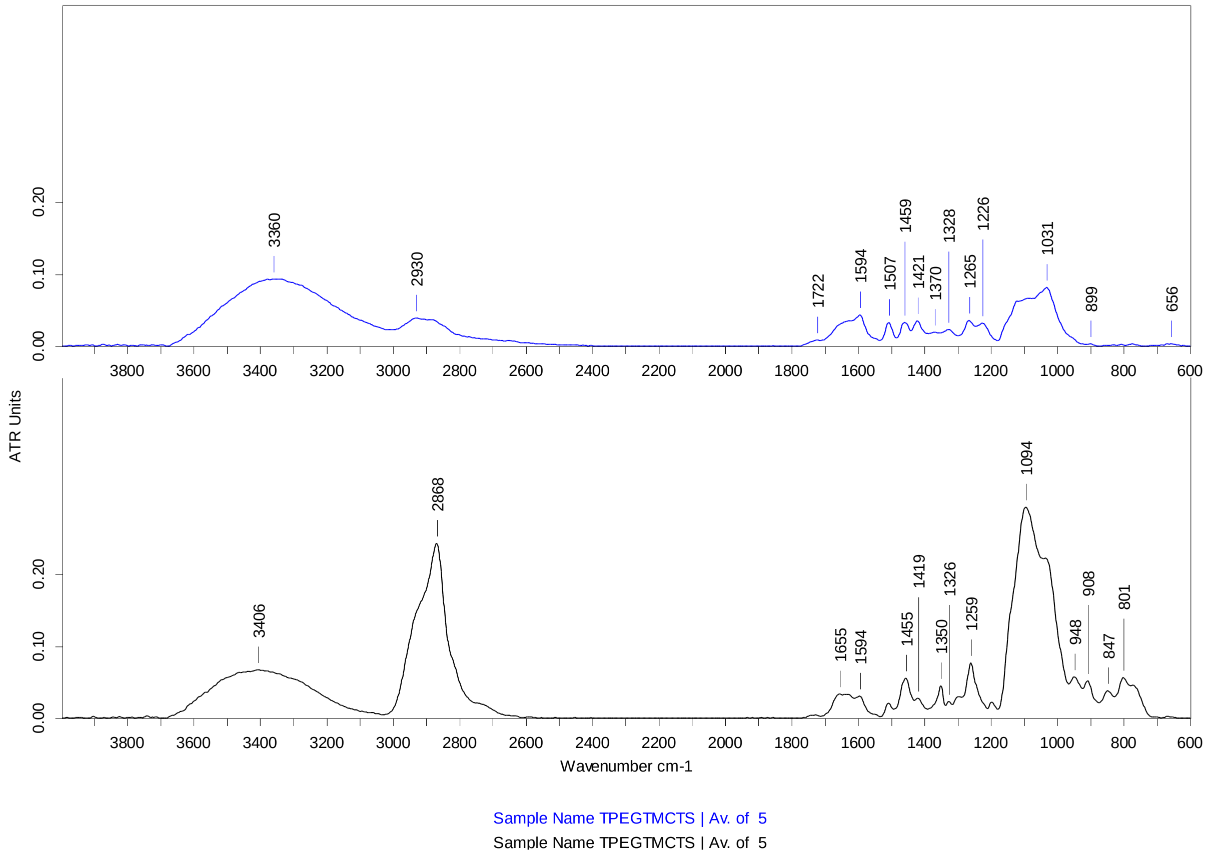

Fig. S20. Stacked FT-IR spectra of wood sample treated with TPEGTMCTS before (black) and after soaking (blue)

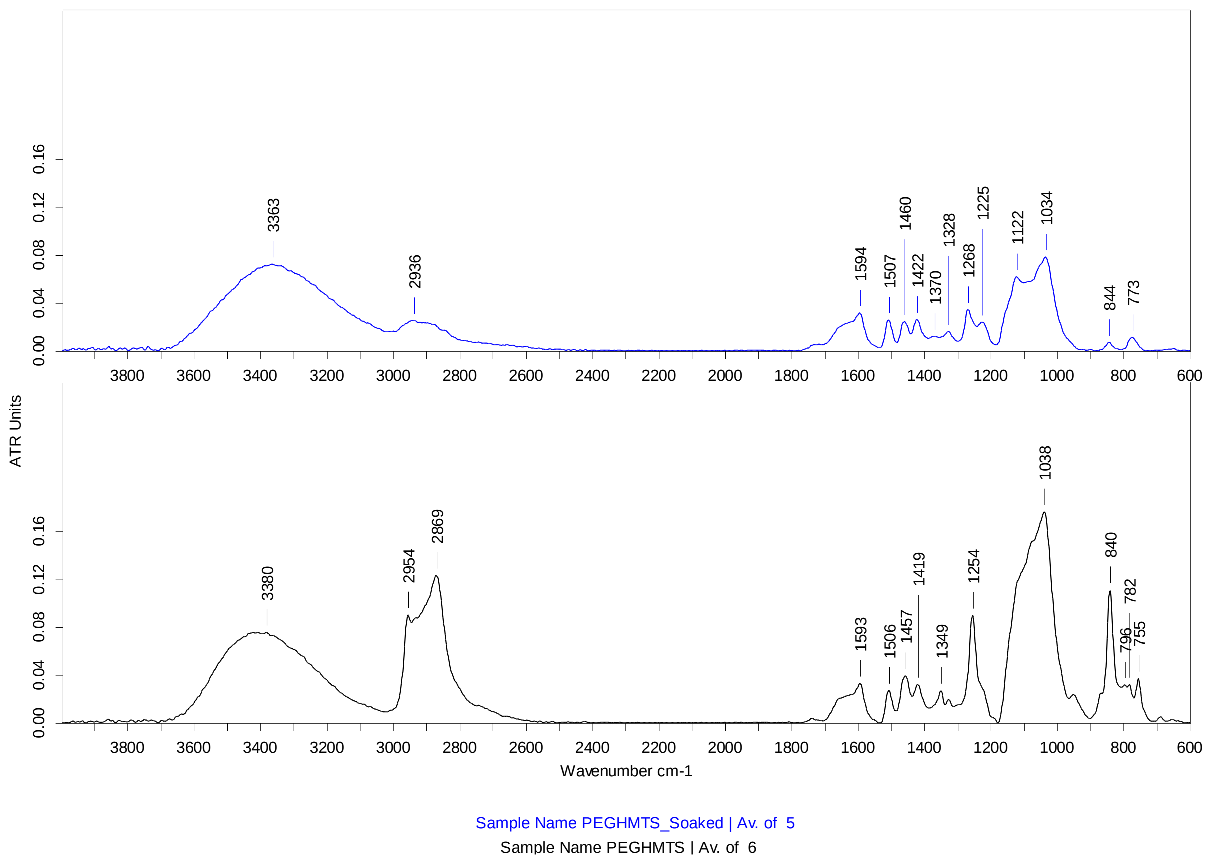

Fig. S21. Stacked FT-IR spectra of wood sample treated with PEGHMTS before (black) and after soaking (blue)

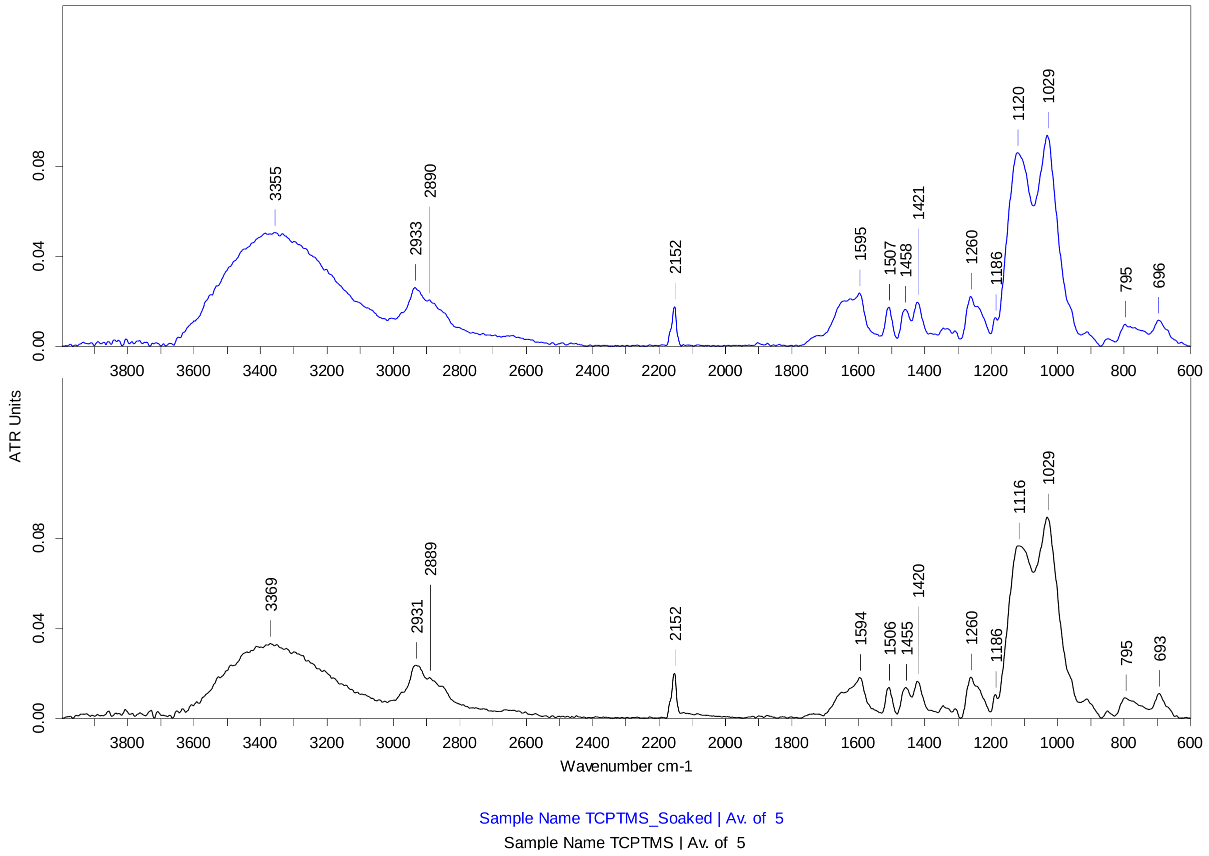

Fig. S22. Stacked FT-IR spectra of wood sample treated with TCPTMS before (black) and after soaking (blue)

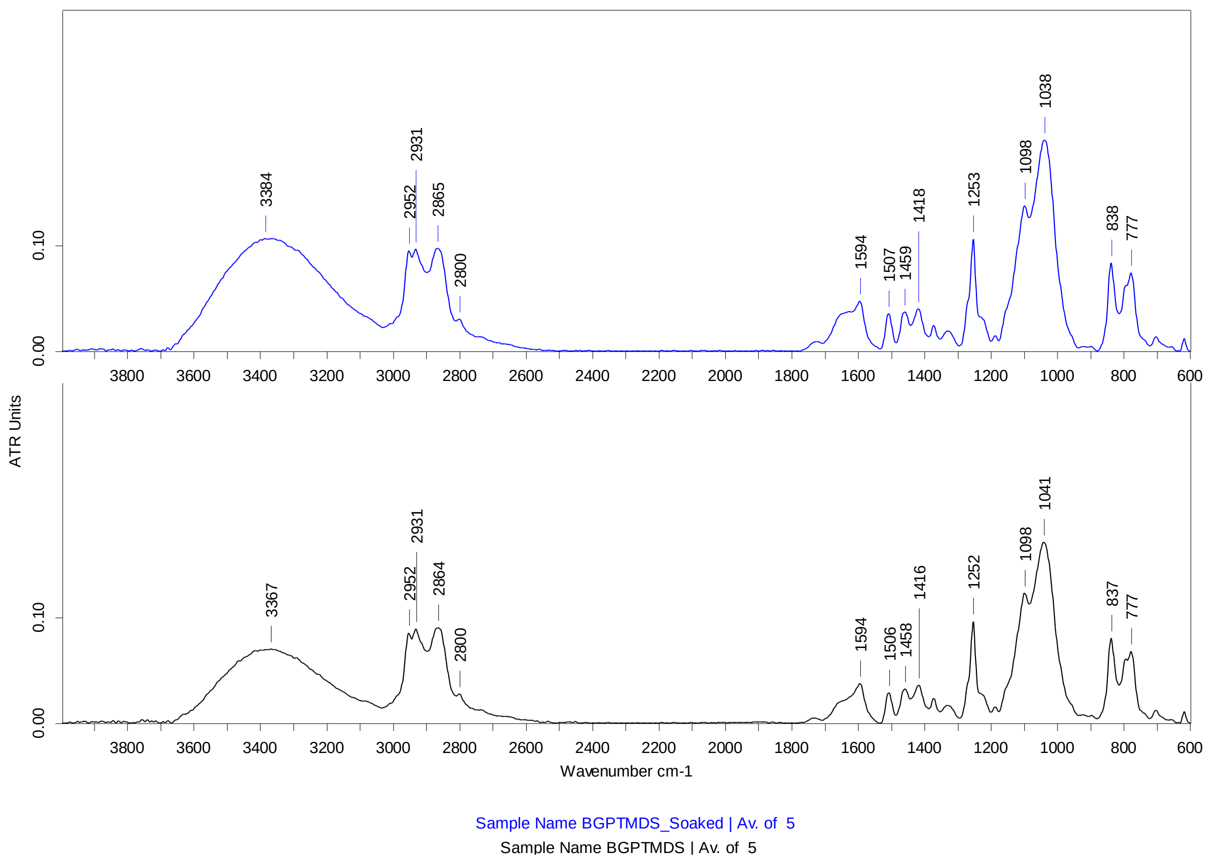

Fig. S23. Stacked FT-IR spectra of wood sample treated with BGPTMDS before (black) and after soaking (blue)

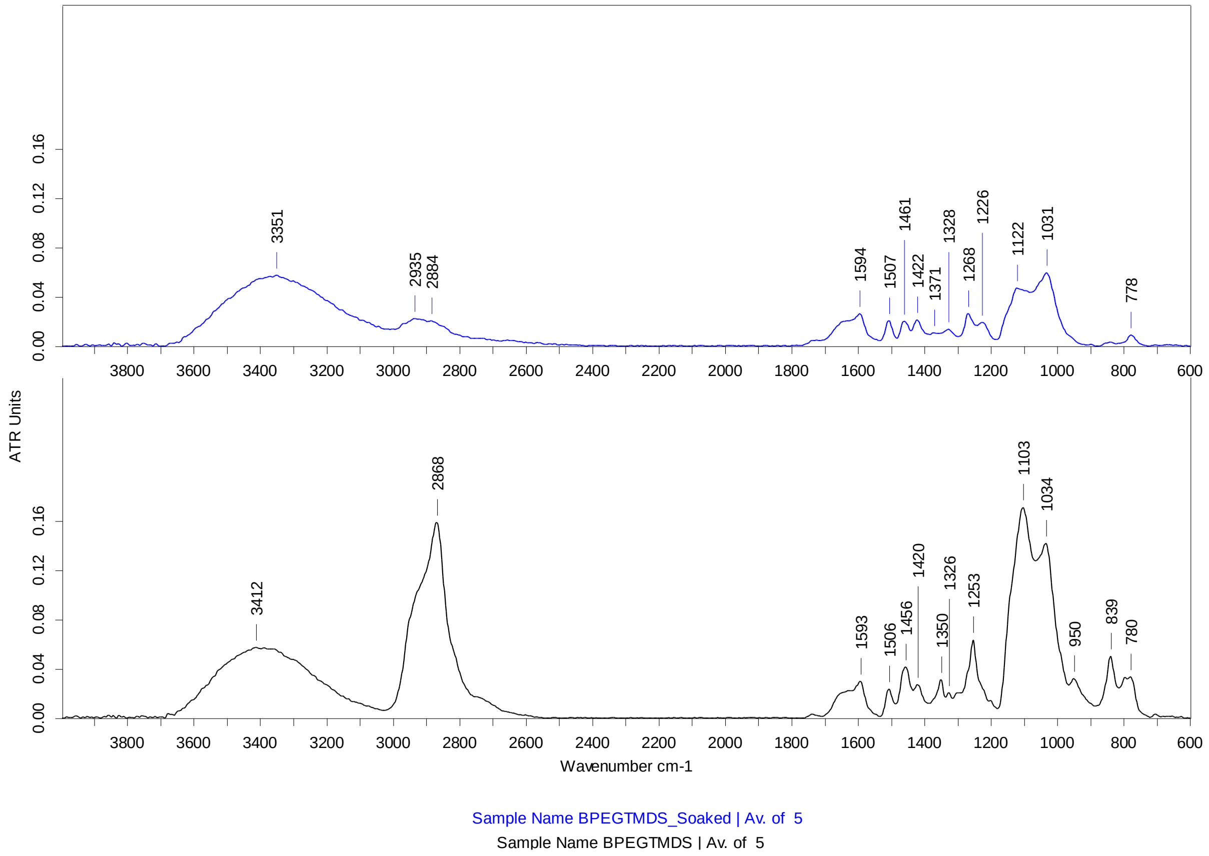

Fig. S24. Stacked FT-IR spectra of wood sample treated with BPEGTMDS before (black) and after soaking (blue)

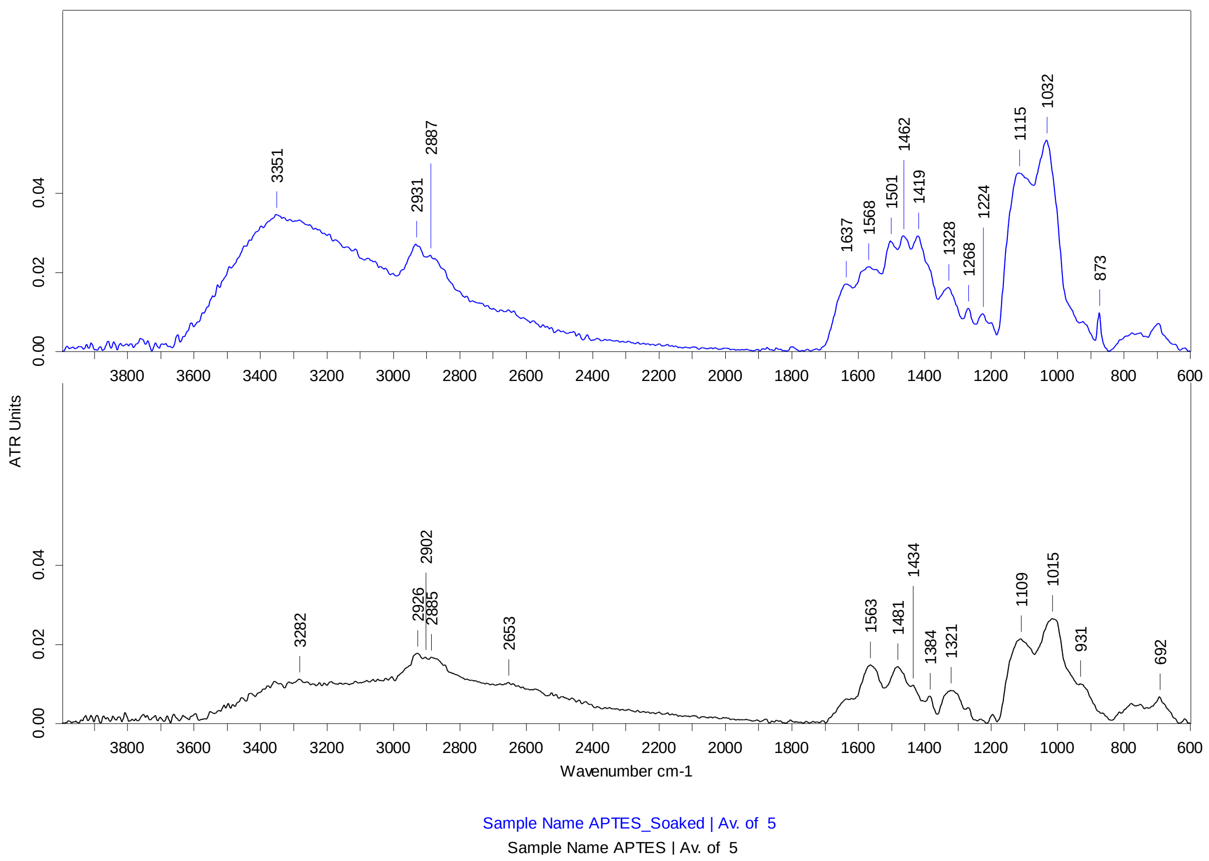

Fig. S25. Stacked FT-IR spectra of wood sample treated with APTES before (black) and after soaking (blue)

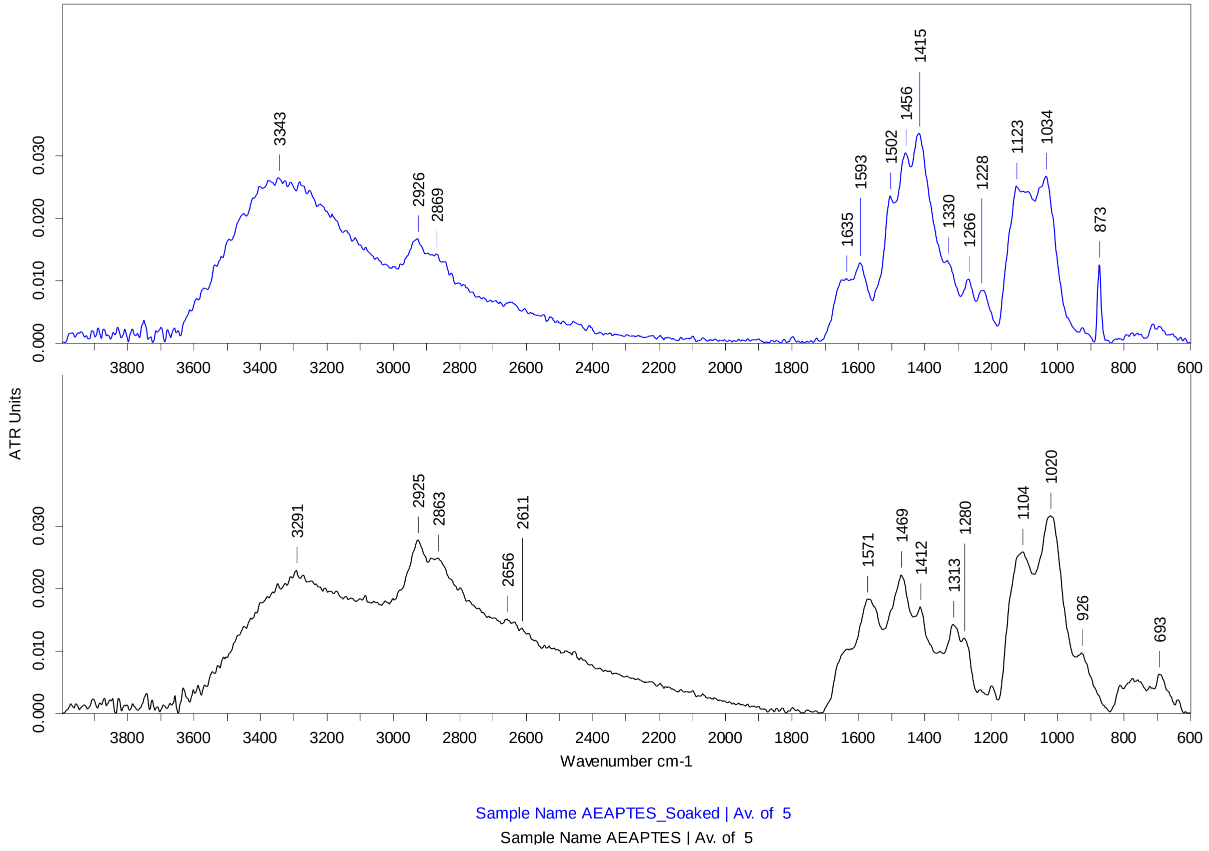

Fig. S26. Stacked FT-IR spectra of wood sample treated with AEAPTES before (black) and after soaking (blue)

Selected SEM-EDX images of cross sections through the middle of samples with Si mapping:

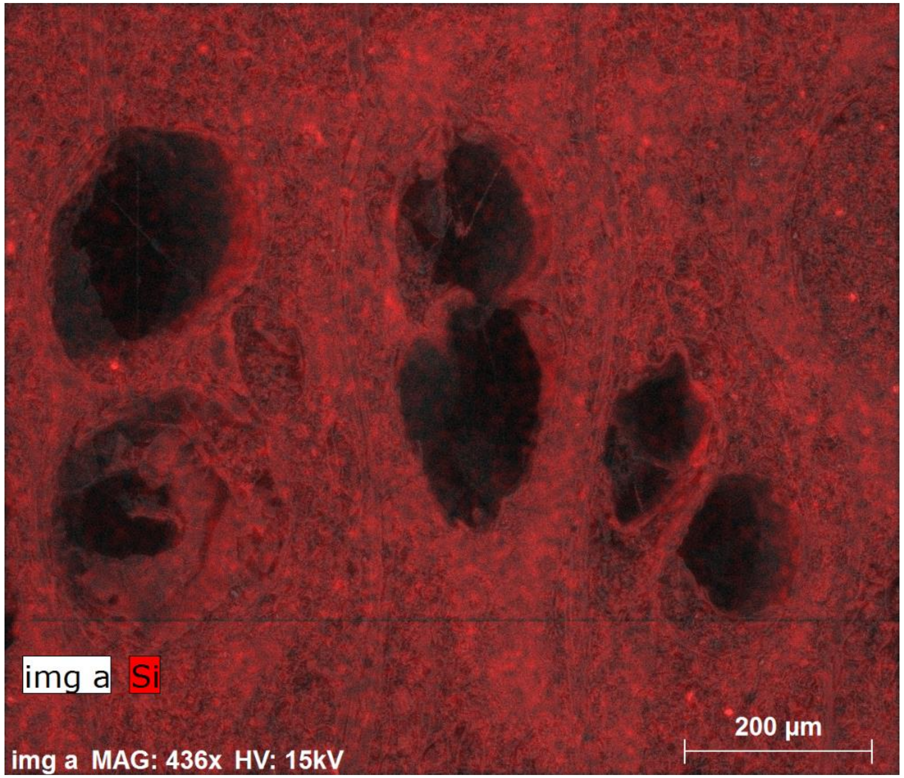

Fig. S27. SEM-EDX image of MTMS-treated sample

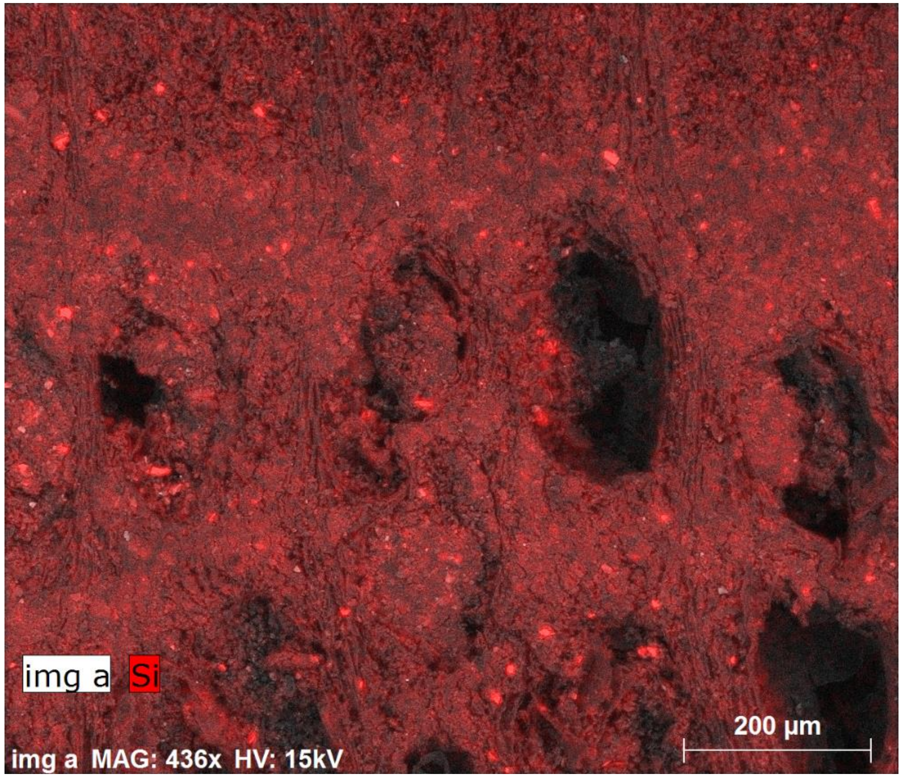

Fig. S28. SEM-EDX image of OTMS-treated wood

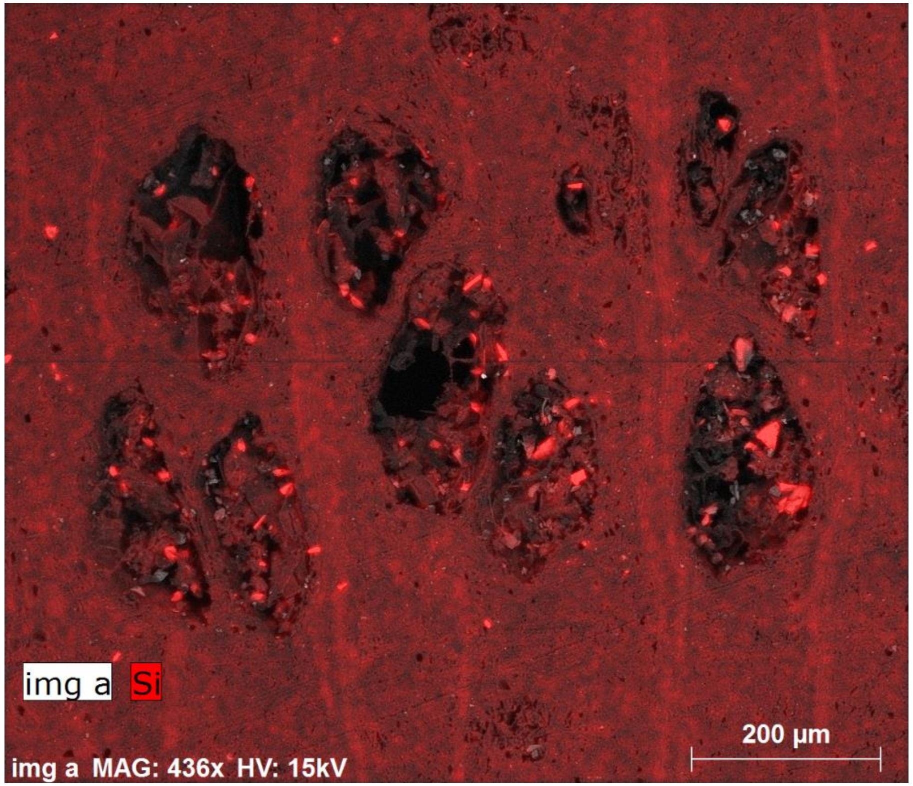

Fig. S29. SEM-EDX image of BAPTMDS -treated wood

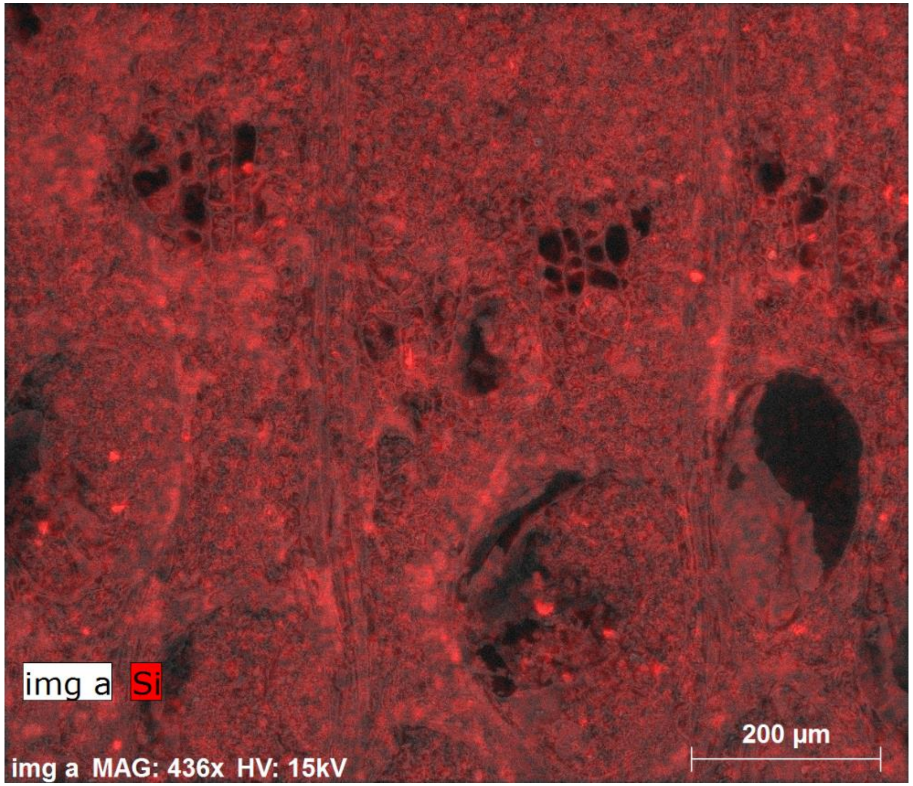

Fig. S30. SEM-EDX image of MPTMS-treated wood

References:

53. Al-Oweini, R., & El-Rassy, H. Synthesis and characterization by FTIR spectroscopy of silica aerogels prepared using several Si (OR) 4 and R″ Si (OR′) 3 precursors. *J. Mol. Struct.* **919**(1-3), 140-145 (2009).

54. Benmouhoub, C., Gauthier-Manuel, B., Zegadi, A., & Robert, L. A Quantitative Fourier Transform Infrared Study of the Grafting of Aminosilane Layers on Lithium Niobate Surface. *Appl. Spectrosc.* **71**(7), 1568-1577 (2017).

55. Kavale, M. S., Mahadik, D. B., Parale, V. G., Wagh, P. B., Gupta, S. C., Rao, A. V., & Barshilia, H. C. Optically transparent, superhydrophobic methyltrimethoxysilane based silica coatings without silylating reagent. *Appl. Surf. Sci.* **258**(1), 158-162 (2011).

56. Latthe, S. S., Imai, H., Ganesan, V., & Rao, A. V. Porous superhydrophobic silica films by sol–gel process. *Micropor. Mesopor. Mat.* **130**(1-3), 115-121 (2010).

57. Lin, J., Chen, H., Fei, T., & Zhang, J. Highly transparent superhydrophobic organic–inorganic nanocoating from the aggregation of silica nanoparticles. *Colloids Surf., A* **421**, 51-62 (2013).

58. Matias, T., Varino, C., de Sousa, H. C., Braga, M. E., Portugal, A., Coelho, J. F., & Durães, L. Novel flexible, hybrid aerogels with vinyl-and methyltrimethoxysilane in the underlying silica structure. *J. Matter Sci.* **51**(14), 6781-6792 (2016).

59. Pasteur, G. A., & Schonhorn, H. Interaction of Silanes with Antimony Oxide to Facilitate Particulate Dispersion in Organic Media and to Enhance Flame Retardance. *Appl. Spectrosc.* **29**(6), 512-517 (1975).

60. Popescu, C. M., Popescu, M. C., & Vasile, C. Characterization of fungal degraded lime wood by FT-IR and 2D IR correlation spectroscopy. *Microchem. J.* **95**(2), 377-387 (2010).

61. Popescu, M. C., Froidevaux, J., Navi, P., & Popescu, C. M. Structural modifications of Tilia cordata wood during heat treatment investigated by FT-IR and 2D IR correlation spectroscopy. *J. Mol. Struct.* **1033**, 176-186 (2013).

62. Gun'ko, V. M., Turov, V. V., Krupska, T. V., Protsak, I. S., Borysenko, M. V., & Pakhlov, E. M. Polymethylsiloxane alone and in composition with nanosilica under various conditions. *J. Colloid Interface Sci.* **541**, 213-225 (2019).

63. Sifuentes-Nieves, I., Hernández-Hernández, E., Neira-Velázquez, G., Morales-Sánchez, E., Mendez-Montealvo, G., & Velazquez, G. Hexamethyldisiloxane cold plasma treatment and amylose content determine the structural, barrier and mechanical properties of starch-based films. *Int. J. Biol. Macromol.* **124**, 651-658 (2019).

64. Stuart, B. H. Infrared Spectroscopy: Fundamentals and Applications. John Wiley & Sons, Ltd. doi: 10.1002/0470011149 (2004).
